# Analysis of *Dendrobium huoshanense* transcriptome unveils putative genes associated with active ingredients synthesis

**DOI:** 10.1186/s12864-018-5305-6

**Published:** 2018-12-29

**Authors:** Yingdan Yuan, Maoyun Yu, Zhaohui Jia, Xue’er Song, Yingquan Liang, Jinchi Zhang

**Affiliations:** 1 Co-Innovation Center for Sustainable Forestry in Southern China, Nanjing Forestry University, Nanjing, 210037 China; 2 Jiangsu Province Key Laboratory of Soil and Water Conservation and Ecological Restoration, Nanjing Forestry University, Nanjing, 210037 China; 3 Anhui Tongjisheng Biotechnology Co., Ltd, Lu’an, 237000 China; 4 Cultivation and Industrialization Center of Rare Medicinal Plants in Ta-pieh Mountains, West Anhui University, Lu’an, 23700 China; 5 Institute of Pathology and Southwest Cancer Center, The First Affiliated Hospital, Third Military Medical University, Chongqing, 400038 China

**Keywords:** *Dendrobium huoshanense*, Transcriptome, Polysaccharides synthesis, Alkaloids synthesis, Glycosyltransferase

## Abstract

**Background:**

*Dendrobium huoshanense* C.Z. Tang et S.J. Cheng is a traditional Chinese herbal medicine with high medicinal value in China. Polysaccharides and alkaloids are its main active ingredients. To understand the difference of main active ingredients in different tissues, we determined the contents of polysaccharides and alkaloids in the roots, stems and leaves of *D. huoshanense*. In order to explore the reasons for the differences of active ingredients at the level of transcription, we selected roots, stems and leaves of *D. huoshanenese* for transcriptome sequencing and pathway mining.

**Results:**

The contents of polysaccharides and alkaloids of *D. huoshanense* were determined and it was found that there were significant differences in different tissues. A total of 716,634,006 clean reads were obtained and 478,361 unigenes were assembled by the Illumina platform sequencing. We identified 1407 carbohydrate-active related unigenes against CAZy database including 447 glycosyltransferase genes (GTs), 818 glycoside hydrolases (GHs), 60 carbohydrate esterases (CEs), 62 carbohydrate-binding modules (CBMs), and 20 polysaccharide lyases (PLs). In the glycosyltransferases (GTs) family, 315 differential expression genes (DEGs) were identified. In total, 124 and 58 DEGs were associated with the biosynthesis of alkaloids in Dh_L vs. Dh_S and Dh_R vs. Dh_L, respectively. A total of 62 DEGs associated with the terpenoid pathway were identified between Dh_R and Dh_S. Five key enzyme genes involved in the terpenoids pathway were identified, and their expression patterns in different tissues was validated using quantitative real-time PCR.

**Conclusions:**

In summary, our study presents a transcriptome profile of *D. huoshanense*. These data contribute to our deeper relevant researches on active ingredients and provide useful insights into the molecular mechanisms regulating polysaccharides and alkaloids in *Dendrobium*.

**Electronic supplementary material:**

The online version of this article (10.1186/s12864-018-5305-6) contains supplementary material, which is available to authorized users.

## Background

*Dendrobium* is the second largest genus of the Orchidaceae widely distributed in tropical and subtropical regions of Asia, Oceania, and other areas [1]. *Dendrobium huoshanense* C.Z. Tang et S.J. Cheng is a perennial epiphytic herb, which belongs to Orchidaceae *Dendrobium* Sw. The wild *D. huoshanense* is endangered because of over-exploitation and habitat deterioration. It is distributed only in the Ta-pieh Mountains of China including Huoshan, Jinzhai, Yuexi and Shucheng County in Anhui Province and Yingshan County in Hubei Province. *D. huoshanense* has been used for nourishing the stomach, promoting the secretion of body fluids as well as treating throat inflammation and enhancing immunity in China [2]. The active medicinal components of *D. huoshanense* are very complex, including polysaccharides, alkaloids, amino acids, phenols, coumarins, terpenes, flavonoids, benzyl compounds, and several trace mineral elements [3, 4], and its major components are polysaccharides and alkaloids [5, 6].

Previous work focused on structural identification and characterization of polysaccharides and alkaloid components of *Dendrobium* species. *Dendrobium* polysaccharides are mainly composed of glucose, galactose, xylose, rhamnose, mannose and other monosaccharides. However, monosaccharide components and contents of polysaccharides differ among *Dendrobium* species [7, 8]. Polysaccharides are natural macromolecules with anti-tumor, anti-oxidation, anti-aging, antibacterial, antiviral, hypoglycemic, blood lipid metabolism, anti-radiation, anticoagulant and other biological activities [9–11]. The polysaccharide HPS-1B23 was isolated from *D. huoshanense* by chemical methods and the nuclear magnetic resonance (NMR) technique. It was composed of glucose, mannose and galactose, and the molar ratio of each monosaccharide was 31:10:8 [12]. A new *Dendrobium* polysaccharide DHPD1 with a molecular weight of 3.2 × 10^3^ Da was isolated from the protocorm of *D. huoshanense*, and the results showed that it was mainly composed of glucose, arabinose and galactose at a molar ratio of 1.023: 0.023: 0.021 and small amounts of mannose and xylose [13]. A water-soluble polysaccharide was isolated and purified from the fresh stems of *D. huoshanense*, which mainly consisted of glucose, xylose, and galactose in molar ratio of 1.1:1.0:0.5, as well as trace of galacturonic acid [14]. Above all, the main monosaccharides of the *Dendrobium* species were glucose and mannose. Now, most studies of polysaccharides are related to their structure, composition and bioactivity, but there are few studies that focus on the key enzymes and genes in the biosynthesis of polysaccharide. Therefore, it is important to study the molecular mechanism of polysaccharide synthesis in *D. huoshanense*.

The contents, structures and pharmacological effects of *Dendrobium* alkaloids have already been clarified. In 2000, Morita et al. isolated three new *Dendrobium*-type sesquiterpenoid alkaloids from the whole plants of *Dendrobium* Snowflake [15]. Through the study of the anti-inflammatory mechanism of total alkaloids from *D. nobile*, we found that the alkaloid enriched extract from *D. nobile* attenuated lipopolysaccharide-induced hyperphosphorylation of tau protein in rat’s hippocampus and protected against lipopolysaccharide-induced apoptosis in rat brain [16]. Bioactive components and pharmacological effects play important roles in the study of medicinal plants. Therefore, biosynthetic pathway analysis and key enzyme gene mining have also become the main aim of transcriptome research in medicinal plants. Unveiling the *D. officinale* transcriptome allowed discovery of alkaloids that belong to the terpenoid indole alkaloid class, and to identify five key enzymes involved in the construction of the backbone of terpene indole alkaloids [17]. It is known that the mevalonate (MVA) pathway and the 2-C-methyl-D-erythritol 4-phosphate (MEP) pathway supply the prenyl diphosphates in plants [18].

So far, several *D. officinale* transcriptomes [17, 19, 20] and a few genomes [21, 22] have been sequenced. A lot of key enzyme-encoding genes involved in the synthesis and metabolic pathways have been identified. However, there are some differences in the genomes of *D. huoshanense* and *D. officinale*, and *D. huoshanense* that has not been subjected to genome sequencing, so there are few studies on *D. huoshanense*. Here, we measured the content of polysaccharides and alkaloids in the roots, stems and leaves of *D. huoshanense*. It was found that the contents of polysaccharides and alkaloids were significantly different in different tissues. In order to explore the causes of these differences, we collected the roots, stems and leaves of *D. huoshanense* for transcriptome sequencing. Through comparative analysis of different tissues, we obtained a large number of differentially expressed genes, including key genes and transcription factors involved in the synthesis of polysaccharides and alkaloids. Furthermore, we identified and analyzed the key metabolic pathways MVA and MEP involved in the synthesis of alkaloids from *D. huoshanense*. The expression levels of key genes on the MVA and MEP metabolic pathways were further verified by real-time quantitative PCR. These results provide sufficient data resources and new insights for further study of the molecular mechanisms of the synthesis of polysaccharides and alkaloids from *D. huoshanense*.

## Methods

### Plant materials

*D. huoshanense* plants were artificially cultivated and collected from the greenhouse of Anhui Tongjisheng Biotechnology Company, Lu’an, China. Seed germination and protocorm-like bodies growth were cultured on half-strength Murashige and Skoog (MS) medium [23] adding 6-BA 0.1 mg·L^− 1^, NAA 0.5 mg·L^− 1^ and 1% additives (30 g·L^− 1^ sucrose + 4 g·L^− 1^ agar + 20% potato) under a 12/12 h light–dark cycle (approx. 30 μmol m^− 2^·S^− 1^) at 25 ± 2 °C. After 6 months, the plants were transplanted into pots and placed in the greenhouse at a temperature of 25–27 °C with a light/dark cycle of 12/12 h and 60–70% relative humidity. Roots, stems and leaves were collected from 2-year-old *D. huoshanense* plants for RNA extraction and determination of polysaccharides and total alkaloids content in March, 2017. In the determination of polysaccharides and total alkaloids, we selected the roots, stems and leaves of five independent plants for determination, that is, five biological replicates. In the transcriptome sequencing, we did three biological replicates of samples from independent plants. All *D. huoshanense* samples were stored at − 80 °C in an ultra-low temperature freezer.

### Determination of polysaccharide and total alkaloid contents

Leaf, stem and root samples were collected from 2-year-old *D. huoshanense* at maturation stage. The phenol-sulfuric acid method was applied to determine the polysaccharide contents in different tissues. The polysaccharide content was determined by using a glucose standard.

The content of total alkaloid was determined using a method that is described as follows. 0.5 g fresh stems were soaked with 5 ml ammonia water, and they were sealed for 30 min. Then 10 ml of chloroform was added and the weight was recorded. And the mixture was in Soxhlet extractor for 2 h with water bath at 75 °C. Then the mixture was cooled and the chloroform was added to the original weight, and then it was filtered. The lower filtrate (1 ml) was placed in a 100-ml volumetric flask and the volume was increased with chloroform. 10 ml sample solution was put in the 50-ml centrifuge tube and 5 ml potassium hydrogen phthalate buffer solution (pH 4.5) was added. Then 1 ml 0.04% bromocresol green solution was added and it was oscillated for 5 min. Then it was placed for 30 min and 5 ml supernviaould like liquid was added in the test tubes and 1 ml 0.01 M NaOH anhydrous ethanol solution was added. The liquid was fully mixed and the absorbance was measured at 620 nm. The total alkaloid content was calculated by using a Dendrobine standard. There were five biological replicates for each tissue in this experiment.

### Total RNA extraction, cDNA library preparation and transcriptome sequencing

Total RNA was extracted using OmniPlant RNA Kit (Cwbio, China) according to the manufacturer’s protocol. The OD260/280 should range from 1.8–2.0 to ensure the purity of the RNA sample. RNA integrity was monitored by agarose gel electrophoresis (1%) and using an Agilent 2100 Bioanalyzer (Agilent Technologies, USA) with RIN number > 7.5. A total of 5 μg RNA was used as an input per sample. Following the manufacturer’s instructions, the samples for transcriptome analysis were prepared using the TruSeqTM RNA Sample Preparation Kit (Illumina, USA). In short, the protocol consisted of the following steps: mRNA was isolated from total RNA using oligo (dT) magnetic beads and cut into short fragments by adding fragmentation buffer. First-strand cDNA was synthesized using random hexamer-primers, taking these short fragments as templates, which were then used to synthesize second-strand cDNA. The products were purified and enriched by PCR to create the final cDNA libraries. The prepared libraries were sequenced on an Illumina Hiseq 2500 platform and 125 bp paired-end reads were generated.

### De novo assembly and functional annotation

To get clean reads, low-quality reads and adapter sequences were removed using SeqPrep (https://github.com/jstjohn/SeqPrep) and Sickle (https://github.com/najoshi/sickle). All clean reads were assembled using Trinity software [24] based on the left.fq and right.fq, with the min_kmer_cov set to 2 and all other parameters set as their defaults. For function annotation, the longest transcript of each gene was defined as the ‘unigene’. Nucleotide sequences of all unigenes were searched against the current version of Nr (NCBI non-redundant protein sequences) [25], GO (Gene ontology) [26], KEGG (Kyoto encyclopedia of genes and genomes) databases [27], and Swiss-Prot (A manually annotated and reviewed protein sequence database) [28], Pfam 32.0 (Protein family) [29] and String 10.0 (Search tool for the retrieval of interacting genes) [30] using BLAST2GO of version 2.5 with a cut-off E-value of 10^− 5^ [31]. In order to identify genes related to carbohydrate activity, all unigenes were searched against CAZy database using BLAST 2.7.1 with a cut-off E-value of 10^− 5^.

### Identification of differentially expressed genes (DEGs)

Gene expression level of all samples was estimated by mapping clean reads to the Trinity transcripts assembly using RSEM version 1.2.15 [32] with the bowtie2 parameter set at mismatch 0. Differential expression analysis of two samples was performed using the edgeR [33]. The thresholds for significant differential expression were a false discovery rate (FDR) < 0.05 and a |log2(fold change)| of ≥1. The identified DEGs were used for GO and KEGG enrichment analyses, which were performed using the Goatools version 0.5.9 (https://github.com/tanghaibao/Goatools) and KOBAS version 2.0.12 with default settings, respectively [34, 35].

### Quantitative real-time PCR (qRT-PCR)

Five genes involved in the MVA and MEP pathways synthesis were analyzed using qRT-PCR. The five key enzyme genes are *FPS*, *HMGR*, *DXR*, *HDR* and *DXS*. All gene IDs and the primer sequences are listed in the Additional file 1: Table S1. The remaining total RNA from transcriptome sequencing was used for qRT-PCR analysis. The specific primers for DEGs were designed by Oligo 7 software. The qRT-PCR was performed on an ABI 7500 Real-time PCR system (Applied Biosystems, USA) using SYBR Premix Ex Taq (Takara, Japan) according to the manufacturer’s protocol. The expressions of *DXR*, *HDR*, *DXS*, *HMGR* and *FPS* were normalized against *Actin* and were calculated by the 2^−ΔΔCT^ method [36]. Pearson correlation analysis was used to calculate the consistency of RNA-seq and qRT-PCR data.

## Results

### Determination of polysaccharides and total alkaloids in three different tissues

**Fig. 1:**
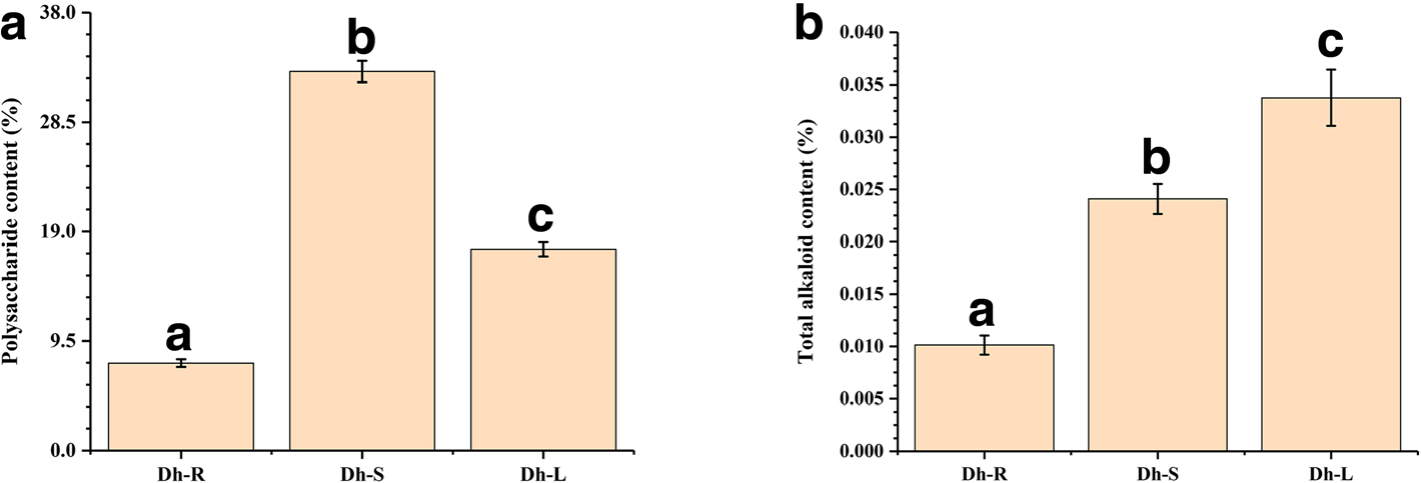
Determination of polysaccharide and total alkaloid contents in different tissues of *D. huoshanense*. **a** Determination of polysaccharide contents in three tissues, including leaf, stem and root. **b** Determination of total alkaloid contents in three tissues, including leaf, stem and root. The a, b, and c letters indicate statistical differences in the same indicator between different tissues, with a significant difference of *p* < 0.05. Error bars represent standard deviations

Polysaccharide and total alkaloids were determined in three different tissues; leaf, stem and root. The results showed that polysaccharide was mainly concentrated in stems, while total alkaloid was at high concentration in leaves. The highest content of polysaccharide was 32.88% for stems and the highest content of total alkaloid was 0.034% for leaves (Fig. 1). However, the content of polysaccharide and total alkaloids is the lowest in the roots (Fig. 1). These results indicate that the polysaccharides and alkaloids of *D. huoshanense* have significant differences in different tissues.

### Transcriptome sequencing and de novo assembly

In this study, nine cDNA libraries (root cDNA libraries: Dh_R1, Dh_R2, Dh_R3; stem cDNA libraries: Dh_S1, Dh_S2, Dh_S3; leaf cDNA libraries: Dh_L1, Dh_L2, Dh_L3) were constructed. The sequencing raw data were deposited in the NCBI Sequence Read Archive (SRA) database under the accession number SRP122499. A total of 736,904,076 reads constituted the raw data file. After trimming the adapter and low-quality sequences, we obtained 716,634,006 clean reads with a total of 105.58 Gb nucleotides. The base average error rate was 0.01%, and the average Q20 and Q30 values were 97.81 and 93.77%, respectively. The average GC content was 47.15% (Table 1).

**Table 1:** Summary of sequencing quality

Sample ID	Raw reads	Clean reads	Clean bases	Error (%)	Q20 (%)	Q30 (%)	GC (%)
Dh_R1	94,007,182	92,489,906	13.68Gb	0.0125	98.18	94.79	46.65
Dh_R2	70,688,878	69,374,122	10.22Gb	0.0131	97.93	94.09	51.27
Dh_R3	85,816,370	84,494,594	12.5Gb	0.0125	98.21	94.83	47.07
Dh_S1	62,673,824	59,985,308	8.78Gb	0.0145	97.34	92.55	46.52
Dh_S2	107,507,880	105,797,304	15.74Gb	0.0116	98.63	95.78	46.34
Dh_S3	71,777,838	68,278,618	9.97Gb	0.0148	97.19	92.26	46.15
Dh_L1	79,875,854	76,612,106	11.22Gb	0.0145	97.39	92.65	46.5
Dh_L2	73,382,858	70,025,838	10.23Gb	0.0148	97.25	92.33	47.05
Dh_L3	91,173,392	89,576,210	13.24Gb	0.0126	98.13	94.64	46.81
Total	736,904,076	716,634,006	105.58Gb				

*Q20* Percentage of bases with a Phred value > 20, *Q30* Percentage of bases with a Phred value > 30, *Error (%)* Base error rate, *GC (%)* Percentage of bases G and C number in the total number of bases

A total of 595,635 transcripts generated; among them, the shortest transcript was 201 bp and the longest transcript was 16,740 bp. The average length was 764.73 bp, and the N50 was 1241 bp. In total, 478,361 unigenes were obtained in the range of 201–16,740 bp with an N50 length of 902 bp (Table 1). Of these, 271,521 unigenes were 200–400 bp, 132,811 unigenes were 400–1000 bp, 53,004 unigenes were 1–2 kb and the remaining 21,025 unigenes were > 2 kb (Table 2).

**Table 2:** Length distribution of unigenes and transcripts

Nucleotide length	Transcripts	Unigenes
200–400 bp	287,366	271,521
401–1000 bp	172,084	132,811
1000–2000 bp	90,507	53,004
> 2000 bp	45,678	21,025
Total	595,635	478,361
Min length (bp)	201	201
Average length (bp)	764.73	611.40
Max length (bp)	16,740	16,740
N50 (bp)	1241	902

### Functional annotation and classification

**Fig. 2:**
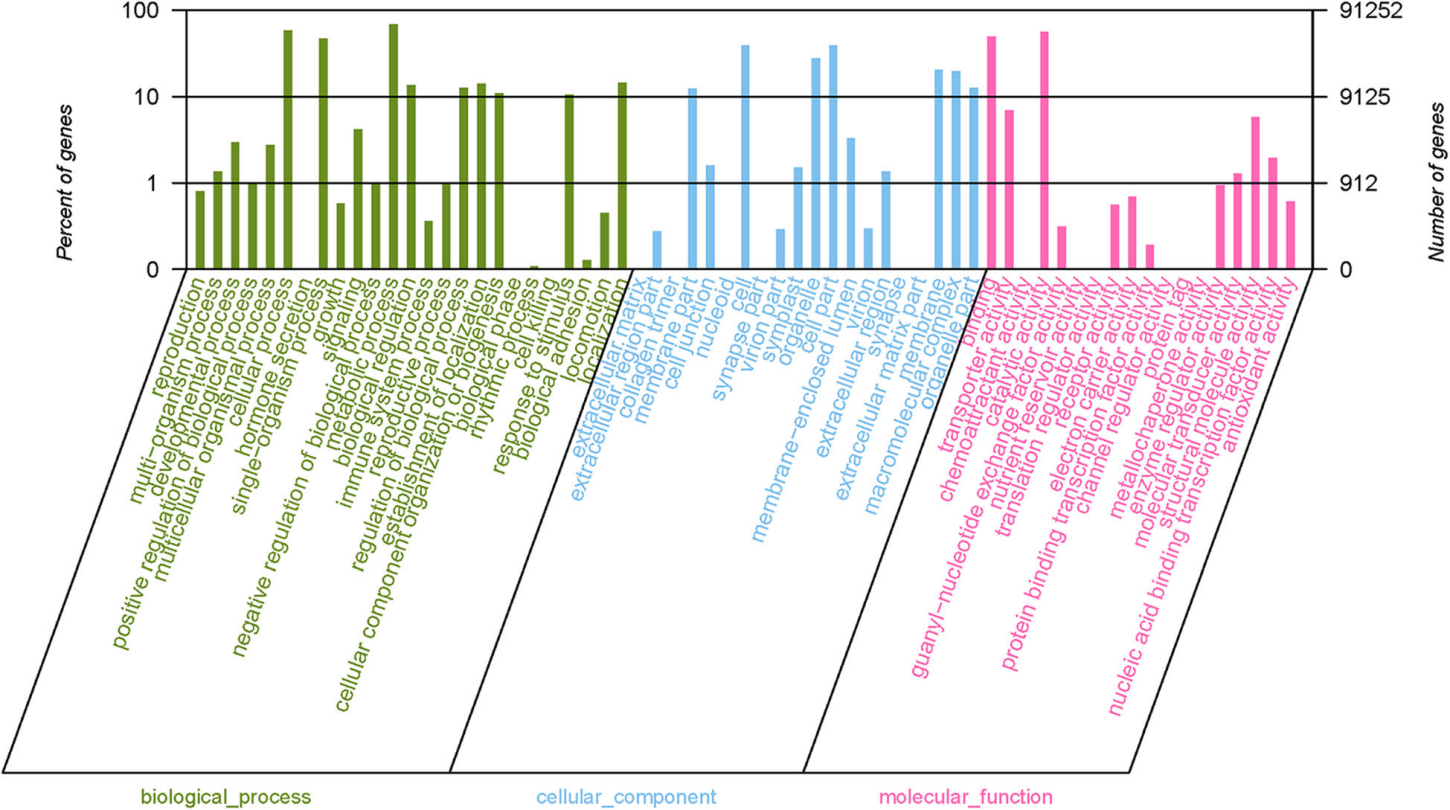
GO classification of unigenes. All the annotated unigenes are divided into three functional GO categories: biological process (BP), cellular component (CC) and molecular function (MF)

**Fig. 3:**
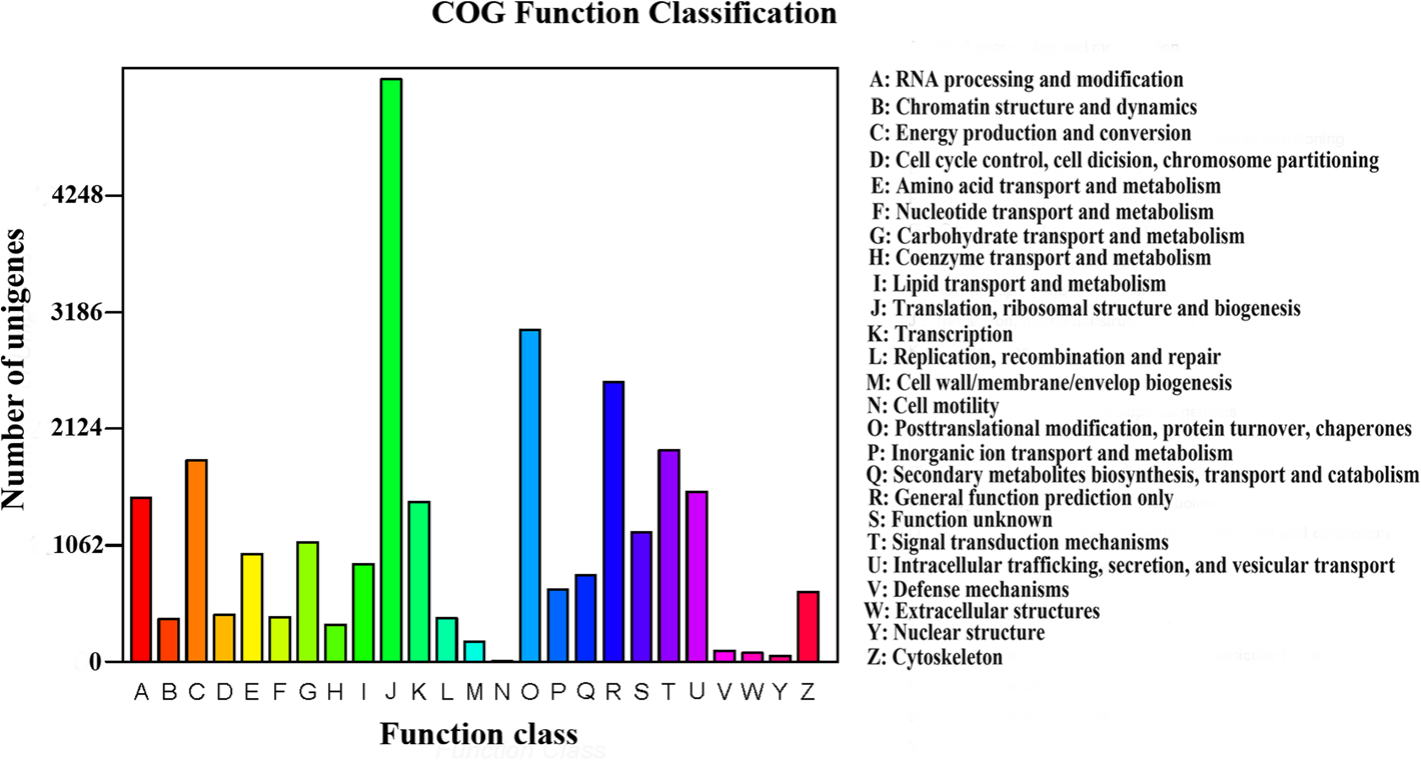
COG annotation of putative proteins. The x-axis indicates the name of the 25 groups of COG. The y-axis indicates the number of unigenes

**Fig. 4:**
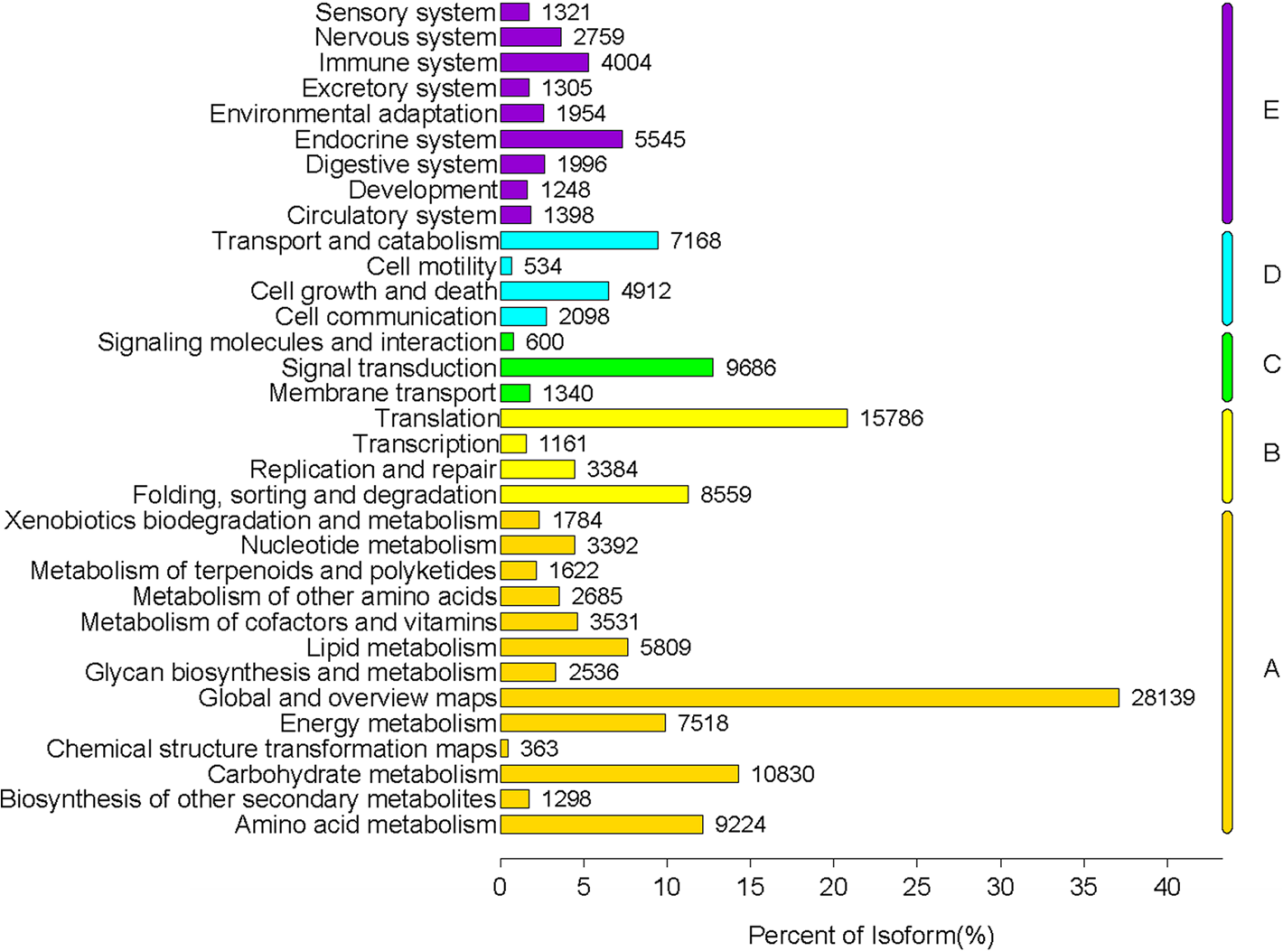
KEGG annotation of putative proteins. The x-axis indicates the percentage of the number of genes annotated to the pathway out of the total number of genes annotated. The y-axis indicates the name of the KEGG metabolic pathway. The genes are divided into five branches according to the KEGG metabolic pathway: Metabolism (**a**), Genetic Information Processing (**b**), Environmental Information Processing (**c**), Cellular Processes (**d**), Organismal Systems (**e**)

Of the total 478,361 unigenes, 196,739, 101,132, 108,417, 69,529 and 37,775 had a hit in the NR, Swiss-Prot, KEGG, Pfam and String database, respectively (Table 3). A total of 14,743 unigenes were annotated to five major databases in common (Additional file 2: Figure S3). A total of 91,252 annotated unigenes were grouped into 63 functional groups by using BLAST2GO [31]. Among these groups, 25 groups were involved in ‘biological processes’ (BP), 20 groups in ‘cellular components’ (CC) and 18 groups in ‘molecular functions’ (MF). Metabolic processes, cellular processes and catalytic activities are important life activities in plants. In *D. huoshanens*e, most of the important active ingredients belong to metabolites such as polysaccharides and alkaloids, and their production depends on the catalysis of key enzymes. Therefore, the three top GO terms were metabolic process, cellular process and catalytic activity (Fig. 2). A total of 27,764 unigenes were annotated to 25 groups in the COG database, the three top terms were J (Translation, ribosomal structure and biogenesis), O (Posttranslational modification, protein turnover, chaperones) and R (General function prediction only) (Fig. 3). In total, 108,417 unigenes annotated to 33 pathways in the KEGG database (Fig. 4). All of the unigenes were divided into five branches according to the KEGG metabolic pathway: Cellular Processes (A); Environmental Information Processing (B), Genetic Information Processing (C), Metabolism (D), and Organismal Systems (E) (Fig. 4). For KEGG, 28,139 unigenes were annotated as the “global and overview maps” pathway, 15,786 unigenes were annotated as the “translation” pathway and 10,830 unigenes were annotated as the “carbohydrate metabolism” pathway (Fig. 4).

**Table 3:** Unigenes annotated to the five databases

Component	Number of unigenes	Percentage (%)
Annotated in Pfam	69,529	14.53%
Annotated in String	37,775	7.90%
Annotated in KEGG	108,417	22.66%
Annotated in Swiss-Prot	101,132	21.14%
Annotated in NR	196,739	41.13%
Total Unigenes	478,361

### Identification of differentially expressed genes (DEGs), GO and KEGG enrichment analysis

**Fig. 5:**
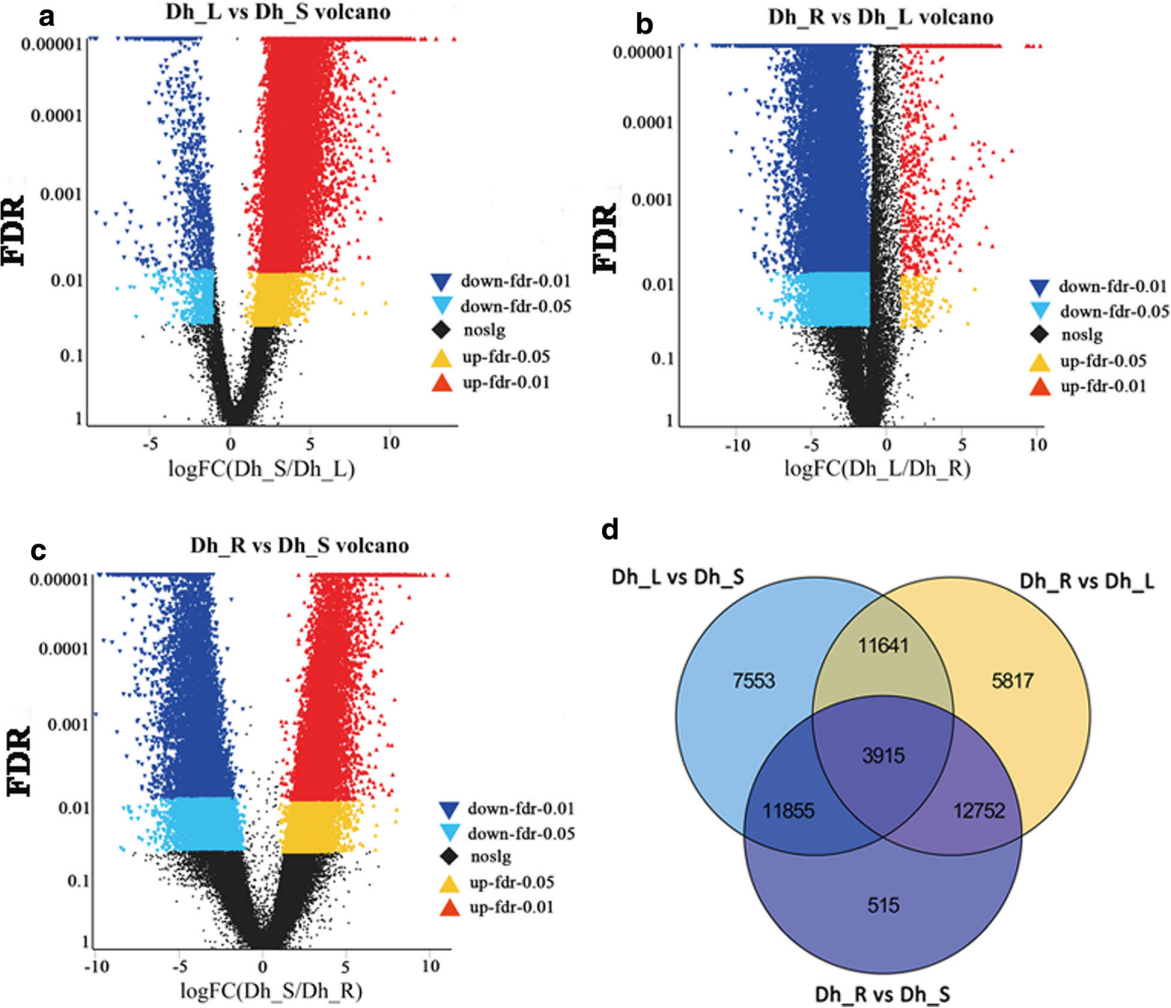
Differentially expressed genes (DEGs) in different comparisons. **a**-**c** Volcano plots of the DEGs in different comparisons. The red dots mean significantly up-regulated genes and the blue dots represent significantly down-regulated genes. The black dots represent non-DEGs. **a** Dh_L vs. Dh_S volcano; **b** Dh_R vs. Dh_L volcano; **c** Dh_R vs. Dh_S volcano. **d** Venn diagram of differentially expressed genes (DEGs) in different comparisons. All DEGs are clustered into three comparison groups represented by three ellipses. The overlapping parts of different ellipses represent the number of DEGs in common from those comparison groups

We used edgeR for examining differential expression of replicated count data. For these DEGs, if FDR < 0.05 and log_2_Fold change ≥1, the DEG was considered as up-regulated but if FDR < 0.05 and log_2_Fold change ≤ − 1, it was considered as down-regulated. There were 34,964 DEGs identified between Dh_L and Dh_S, including 33,763 up-regulated and 1201 down-regulated DEGs (Additional file 3). A total of 34,125 DEGs were identified between Dh_R and Dh_L, including 2206 up-regulated and 31,919 down-regulated DEGs (Additional file 4). There were 29,037 DEGs between Dh_R and Dh_S, 15,352 of which were up-regulated and 13,685 of which were down-regulated (Additional file 5) (Fig. 5a-c). Using a Venn diagram, we compared the three data sets from different comparison groups (Dh_L vs. Dh_S, Dh_R vs. Dh_L and Dh_R vs. Dh_S). In all three comparison groups, 3915 DEGs were identified in common (Fig. 5d). In detail, 11,641 DEGs were identified in both “Dh_L vs. Dh_S” and “Dh_R vs. Dh_L” comparisons; 11,855 DEGs were identified in both “Dh_L vs. Dh_S” and “Dh_R vs. Dh_S” comparisons; while 12,752 DEGs were identified in both “Dh_R vs. Dh_S” and “Dh_R vs. Dh_L” comparisons.

**Fig. 6:**
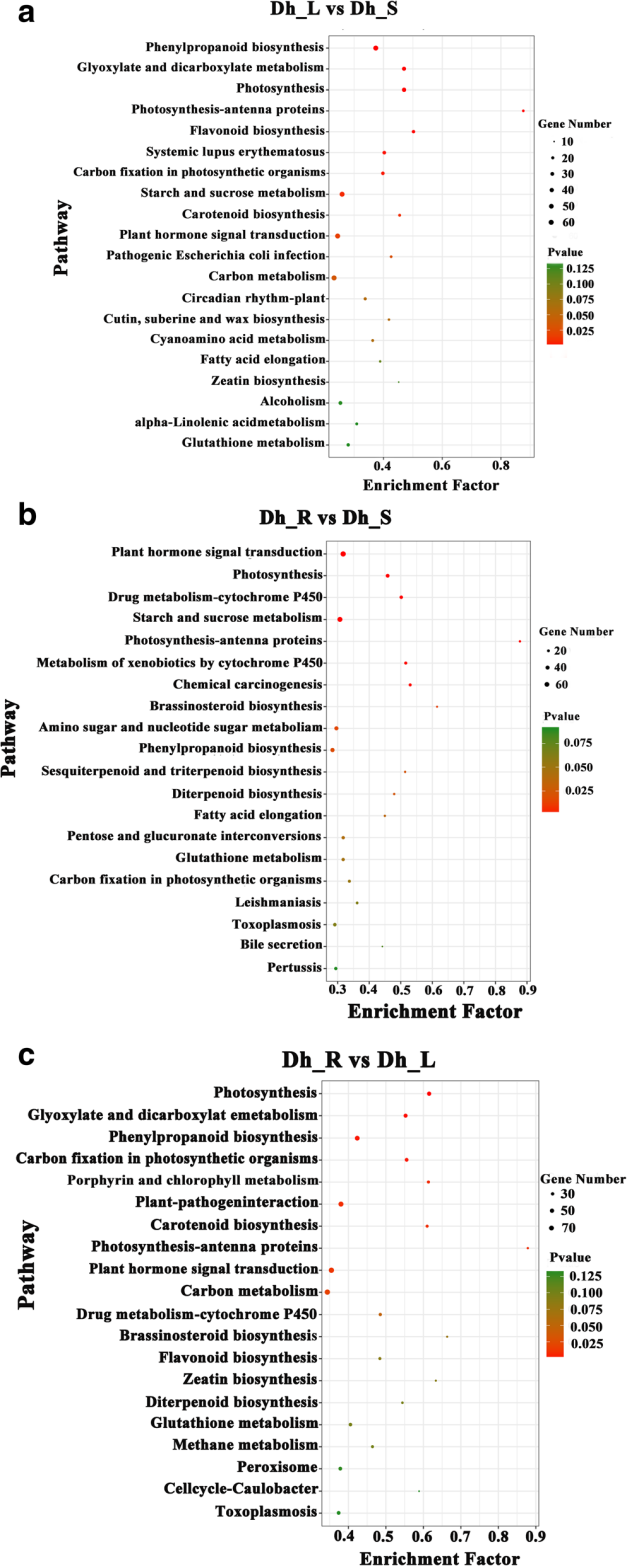
Top 20 of Kyoto Encyclopedia of Genes and Genomes (KEGG) pathway enrichment of DEGs. The x-axis indicates the pathway name, and the y-axis indicates the enrichment factor corresponding to the pathway. The q-value is represented by the color of the dot. The number of DEGs is represented by the size of the dots. **a** Dh_L vs. Dh_S; **b** Dh_R vs. Dh_L; **c** Dh_R vs. Dh_S

To get a better understanding of the DEGs, we performed GO and KEGG enrichment analysis on all DEGs. The GO enrichment of DEGs was conducted by the hypergeometric Fisher exact test, in which *p*-value was calculated and adjusted as corrected p-value. GO terms with corrected p-value < 0.05 were considered to be significantly enriched. For pathway enrichment analysis, all DEGs were assigned to terms in KEGG database and searched for significantly enriched KEGG terms with the same analytic approach. The GO enrichment is shown in Additional file 2: Figure S1 and Additional files 6, 7 and 8. In the KEGG enrichment analysis, the top enriched pathway was flavonoid biosynthesis with 21 DEGs and 37 background unigenes in the Dh_L vs. Dh_S comparison. In the Dh_R vs. Dh_L comparison, the top pathway was diterpenoid biosynthesis with 12 DEGs and 22 background unigenes. Finally, in the Dh_R vs. Dh_S comparison, the top pathway was brassinosteroid biosynthesis with 9 DEGs and 18 background unigenes. The top 20 KEGG enrichment pathways are shown in Fig. 6.

### Identifying *D. huoshanense* carbohydrate-active related genes and DEGs related to polysaccharides

**Fig. 7:**
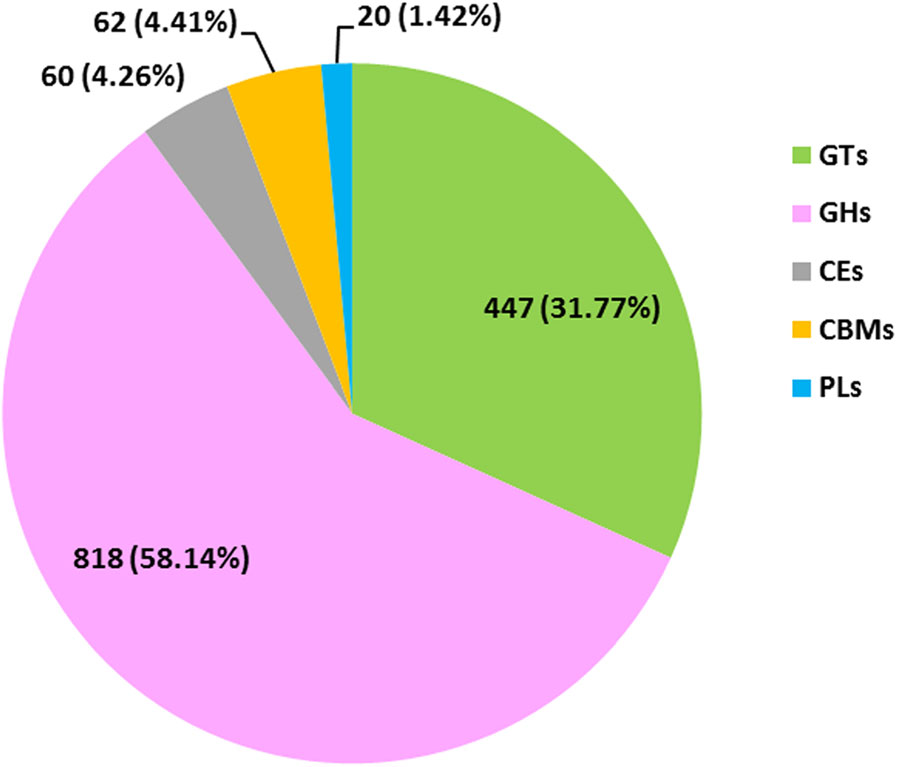
The classification and number of carbohydrate-active enzyme families in *Dendrobium huoshanense* unigenes. GT, Glycosyltransferase; GH, Glycoside Hydrolase; CE, Carbohydrate Esterase; CBM, Carbohydrate-Binding Module; PL, Polysaccharide Lyase

The 1407 carbohydrate-active related unigenes were identified and divided into five gene families (Fig. 7). These unigenes include 447 glycosyltransferase genes (GTs), 818 glycoside hydrolases (GHs), 60 carbohydrate esterases (CEs), 62 carbohydrate-binding modules (CBMs), and 20 polysaccharide lyases (PLs) (Fig. 7). Carbohydrate esterase accounts for the largest share of 58.14%. However, polysaccharide lyases accounts for the smallest, only 1.42%.

**Fig. 8:**
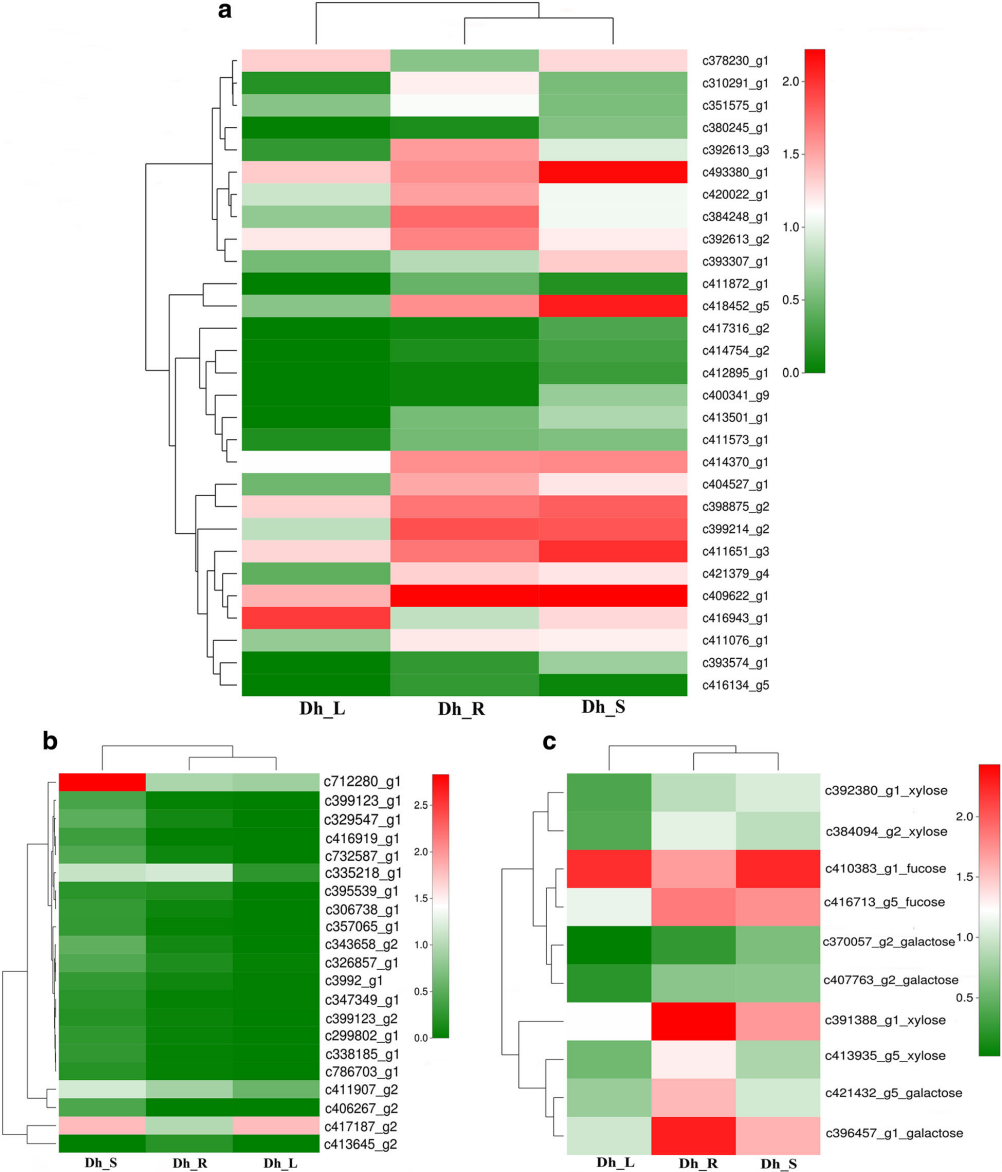
The heatmap of important DEGs associated with polysaccharide. Red indicates high expression genes, while green indicates low expression genes. Color changing from red to green indicate that log10 (FPKM+ 1) gradually changes from big to small. **a** glucosyltransferase (GTF), **b** mannosyltransferase (MNT), **c** fucosyltransferase (FucT), xylosyltransferase (XT) and galactosyltransferase (GAT)

The glycosyltransferases (GTs) are an important and functionally diverse family of enzymes, the main role of GTs in plants is in the biosynthesis of polysaccharides in the plant cell wall [37]. A total of 315 DEGs related to polysaccharide have been identified among these three comparisons. These polysaccharide-related DEGs include 197 GTs, 51 glucosyltransferases (GTFs), 12 fucosyltransferases (FucTs), 34 mannosyltransferases (MNTs), 8 xylosyltransferases (XTs) and 13 galactosyltransferases (GATs) DEGs (Fig. 8 and Additional file 2: Figure S2). In the Dh_L vs. Dh_S comparison, 115 DEGs related to polysaccharides were identified, including 64 up-regulated and 3 down-regulated DEGs of GTs; 22 up-regulated and 1 down-regulated DEGs of GTFs; 2 up-regulated DEGs of FucTs; 19 up-regulated DEGs of MNTs; 3 up-regulated DEGs of XTs and 4 up-regulated DEGs of GATs. In the Dh_R vs. Dh_L comparison, 99 DEGs were identified, including 6 up-regulated and 59 down-regulated of GTs; 2 up-regulated and 14 down-regulated DEGs of GTFs; 2 down-regulated DEGs of FucTs; 1 up-regulated and 6 down-regulated DEGs of MNTs; 1 up-regulated and 3 down-regulated DEGs of XTs; 1 up-regulated and 4 down-regulated DEGs of GATs. In the Dh_R vs. Dh_S comparison, 101 DEGs were identified, including 34 up-regulated and 34 down-regulated DEGs of GTs; 5 up-regulated and 7 down-regulated DEGs of GTFs; 3 up-regulated and 5 down-regulated DEGs of FucTs; 7 up-regulated and 1 down-regulated DEGs of MNTs; 1 up-regulated of XTs and 4 down-regulated DEGs of GATs.

### DEGs related to the biosynthesis of alkaloids in *D. huoshanense*

**Fig. 9:**
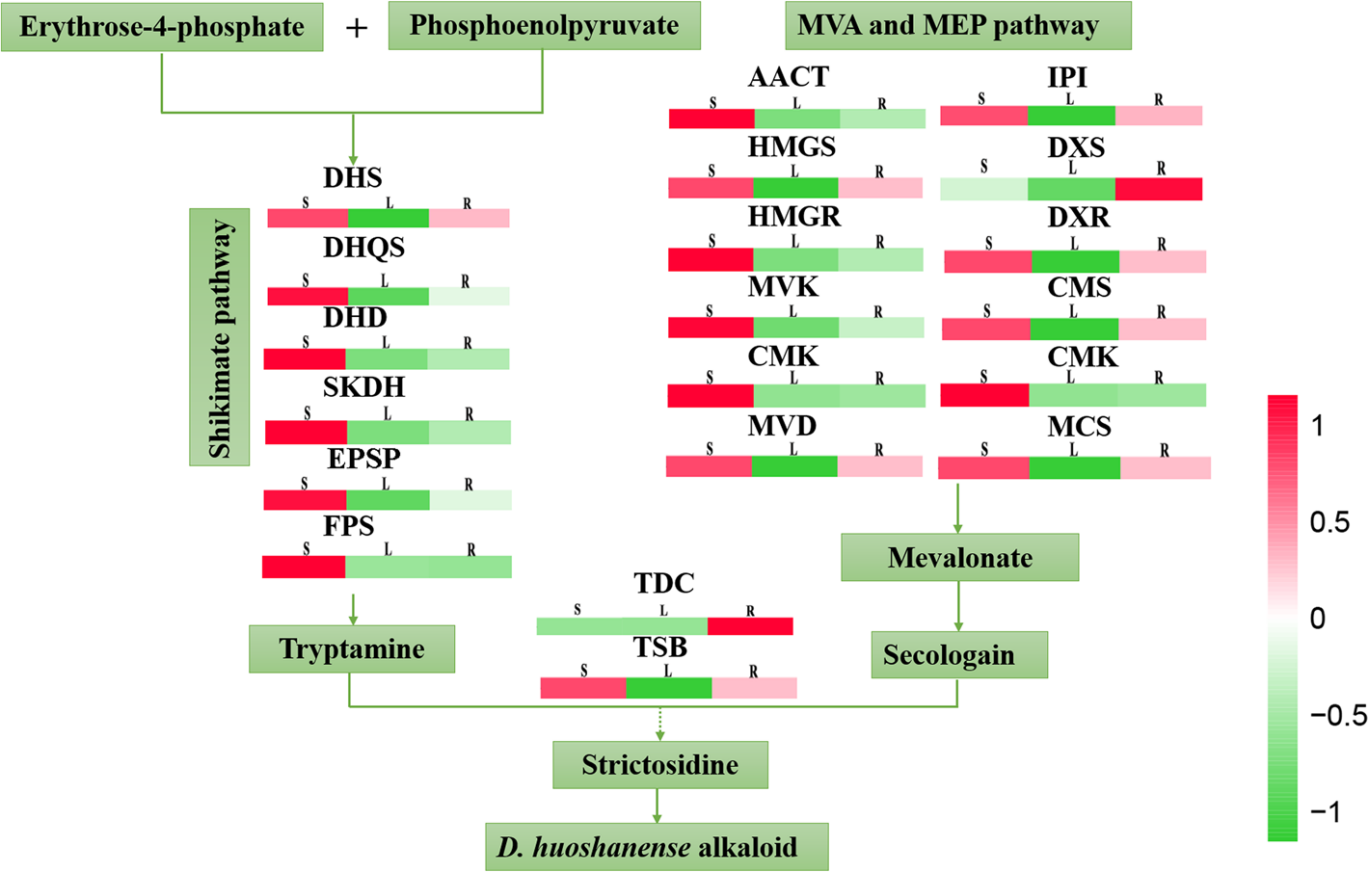
Expression pattern of the unigenes associated with putative alkaloid biosynthesis in *D. huoshanense*. Expression pattern of the unigenes associated with putative upstream elements of alkaloid biosynthetic pathway. Full names of enzymes represented by their abbreviated names were showed in Additional file 1: Table S1. The average expression level of the enzyme encoding unigenes in different tissues is indicated by a heat map. Color changing from red to green indicate that log10 (FPKM+ 1) gradually changes from big to small

At present, some studies have shown that the alkaloids in the genus *Dendrobium* are mostly sesquiterpenoid alkaloids or terpenoid indole alkaloids. Both are derived from the terpenoids pathway (shikimate, MVA or MEP pathways) [17, 20]. DEGs associated with alkaloid biosynthesis in *D. huoshanense* are shown in Additional file 1: Table S1. In total, 53 unigenes associated with six enzymes were targeted to the shikimate pathway, including 3-deoxy-D-arabinoheptulosonate-DHS7-phosphate (DHS), 3-dehydroquinate synthase (DHQS), 3-dehydroquinate acid dehydratase (DHD), shikimate dehydrogenase (SKDH), 5-enolpyruvylshikimate-3-phosphate synthase (EPSP) and fanesyl diphosphase synthase (FPS). Forty-six unigenes associated with 12 enzymes were located to MEP and MVA pathway. What’s more, we identified two key enzyme-encoding genes involved in strictosidine, including tryptophan decarboxylase (TDC) and β-subunit of tryptophan synthase (TSB) (Additional file 9). Based on FPKM of unigenes, the average expression level of the unigenes associated with each enzyme was also determined. Most of encoding enzymes were predominantly expressed in stems, except for TDC and DXS. These two encoding enzymes were highly expressed in roots than stems (Fig. 9).

The downstream synthetic pathway of *D. huoshanense* alkaloid is uncertain due to lack of corresponding compound support. It is speculated that the downstream synthesis pathway is mainly the modification of strictosidine, of which CYP450 may catalyze a subset of the monooxygenase reactions and hydroxylation reactions [38, 39]. In our study, 229 unigenes were identified as putative P450 superfamily members, which are listed in Additional file 1: Table S2. The majority are CYP71 family members (7.8%), followed by CYP3A family members (6.1%) and CYP4 family members (4.8%). According to clan classification, the CYP71 subfamily is likely to be involved in secondary metabolism [40]. Alkaloids are nitrogenous amino acid derivatives whose synthesis requires an aminotransferase enzyme. There were 47 putative unigenes annotated as associated with four independent transaminases (Additional file 1: Table S3).

### Transcription factors involved in *D. huoshanense* transcriptome dataset

Transcription factors (TFs) play an important role in regulating the activity of polysaccharide biosynthesis and other secondary metabolism pathways. By comparison with the TFs from the iTAK database (http://itak.feilab.net/cgi-bin/itak/index.cgi), we identified a total of 2579 expressed TFs from our transcriptome. Each of these TFs belongs to 67 known TF families (Table 4). The most abundant TF family is the zinc finger C2H2 TF including 489 unigenes. The C2H2 TF family is one of the largest family of transcription factors in plants and regulates many biological processes such as plant morphogenesis, transcriptional activation and stress [41]. In addition, some TFs, such as C3H, bHLH, bZIP, MYB-related and WRKY, have been previously demonstrated to play a role in *D. officinale*.

**Table 4:** Transcription factor families identified in the *D. huoshanense* transcriptome dataset

Putative transcription factor family	Number of unique transcripts	Putative transcription factor family	Number of unique transcripts
C2H2	489	TCP	24
C3H	269	SBP	22
bZIP	227	TUB	20
MYB-related	173	HB-HD-ZIP	20
bHLH	145	OFP	20
AP2/ERF-ERF	117	NF-YC	19
C2C2-GATA	94	RWP-RK	19
WRKY	71	E2F-DP	18
CSD	70	LOB	17
HB-other	70	NF-YB	16
NAC	69	PLATZ	15
HSF	66	Tify	14
MYB	64	MADS-MIKC	14
GARP-G2-like	38	C2C2-YABBY	13
GRAS	37	NF-X1	13
LIM	28	B3-ARF	12
FAR1	28	CPP	11
MADS-M-type	28	NF-YA	11
Trihelix	27	zf-HD	10
C2C2-Dof	27	others	108
B3	26	Total number of TFs	2579

### The expression analysis of key enzyme genes by qRT-PCR

**Fig. 10:**
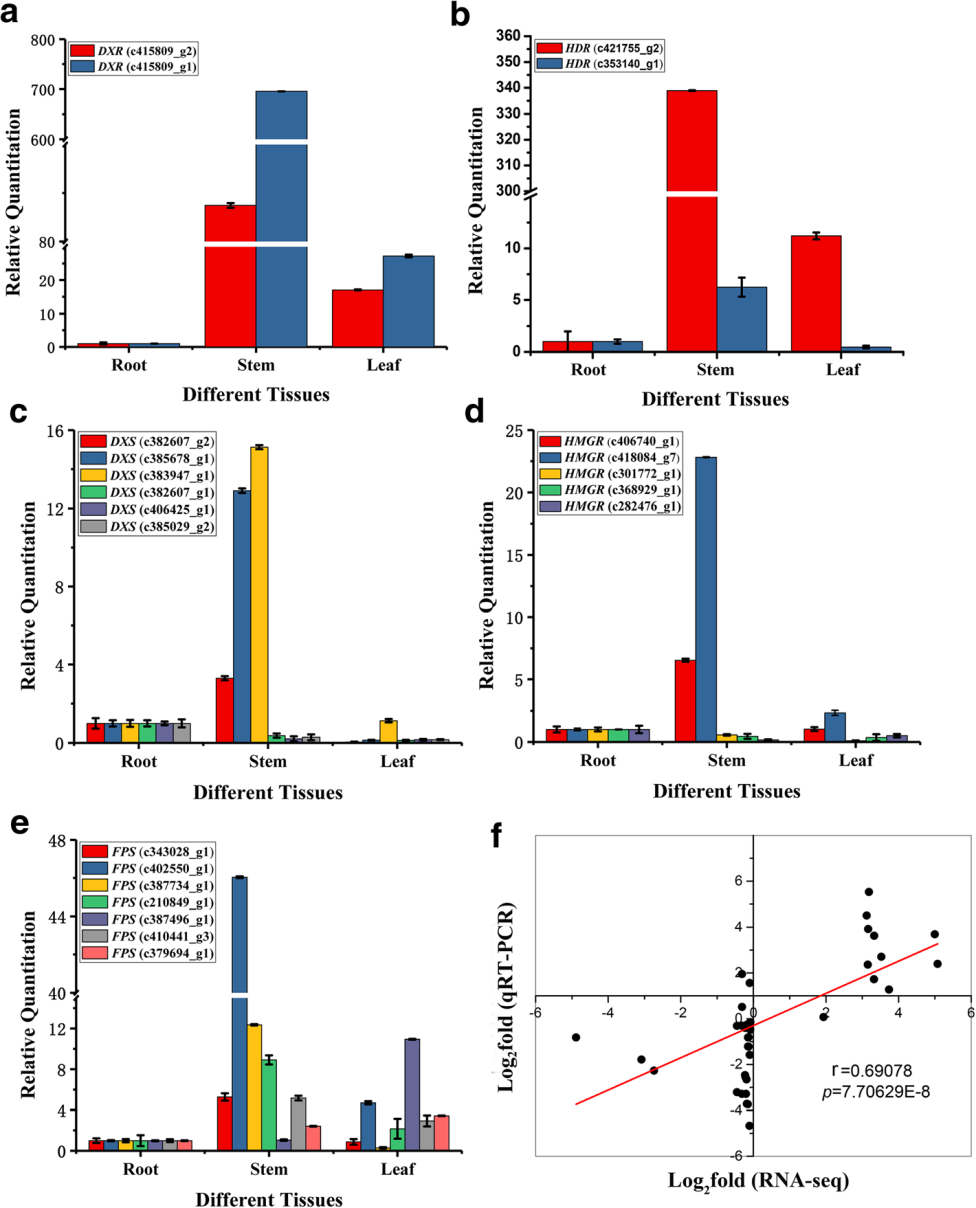
(**a**-**e**) represent the expression patterns of five key enzyme-encoding genes: **a**. *DXR*; **b**. *HDR*; **c**. *DXS*; **d**. *HMGR*; **d**. *FPS*. (**f**) represents the consistency of quantitative expression patterns of the 22 DEGs with the transcriptome data

To validate changes in gene expression patterns, we identified and examined five key enzyme-encoding genes associated with alkaloid biosynthesis including *DXS* (c382607_g2, c385678_g1, c383947_g1, c382607_g1, c406425_g1, c385029_g2), *DXR* (c415809_g2, c415809_g1), *HDR* (c421755_g2, c353149_g1), *HMGR* (c406740_g1, c418084_g7, c301772_g1, c368929_g1, c282476_g1), *FPS* (c343028_g1, c402550_g1, c387734_g1, c210849_g1, c387496_g1, c410441_g3, c379694_g1) using qRT-PCR at the transcriptional level. Primers and sequences are shown in Additional file 1: Table S4. The expression patterns of five key enzyme-encoding genes showed that the highest expression levels of *HDR* and *DXR* were in the stem, while the root material had the lowest expression level. In general, the expression of *FPS* was mostly high in the stem, moderate in leaf material, and relatively low in root material. We found that the transcript level of *HMGR* and *DXS* in root was higher than that in the leaf. However, the levels of *HMGR* and *DXS* expressed in stem were partly higher than those in root. The expression patterns of the 22 DEGs were consistent with the transcriptome data (*r* = 0.69078, *p*-value = 7.70629E-8). These results indicate that our transcriptomic analysis was highly reproducible and reliable (Fig. 10f).

## Discussion

*D. huoshanense* is a traditional Chinese herbal medicine, and its polysaccharides and alkaloids are main medicinal components. At present, a large number of putative polysaccharides and alkaloids biosynthetic genes have been identified in *D. officinale* [17, 19]. In *D. huoshanense*, there are only a few studies on gene cloning, components structure and isolation of polysaccharides and alkaloids. This report is to identify putative genes and metabolic pathways involved in polysaccharides and alkaloid biosynthesis using high-throughput transcriptome sequencing in *D. huoshanense*. In our study, nine libraries of transcriptome data were obtained, which assembled a total of 478,361 unigenes. Using the BLAST algorithm (E-value<1E-5), they were searched against the Nr, KEGG, Swiss-Prot, Pfam and String databases. Many genes involved polysaccharides and alkaloids biosynthesis have been identified and characterized [17, 19]. Our unigene numbers are almost twofold greater than the unigene numbers from a previous study that focused on four different tissues of *D. officinale* [42].

Polysaccharides are the main medicinal ingredients of *D. huoshanense*, and the polysaccharides of *D. huoshanense* are composed of glucose, galactose, mannose, arabinose and rhamnose [3]. Glycosyltransferase (GT) is a class of enzymes that catalyze the transfer of sugar from an active donor to a specific receptor molecule, which is ubiquitous in organisms and forms a superfamily of genes. Glycosyltransferase is involved in development, signal transduction, defense and other biological processes. There are a large number of GT families in *Arabidopsis* and rice (*Oryza sativa*), with more than 450 genes [43–45]. In *D. huoshanense*, a large number of carbohydrate-related DEGs were identified which includes glycosyltransferase, glucosyltransferase, mannosyltransferase, fucosyltransferase, xylosyltransferase and galactosylatransferase. These genes may play important roles in the synthesis of polysaccharides from *D. huoshanens*e. The results showed that most of these genes were expressed more highly in stems than in leaves and roots, which is consistent with previous work [20]. The differences of *D. huoshanens*e and *D. officinale* are not only in carbohydrate content but also in the number of genes related to carbohydrate synthesis. In a previous study, 1081 genes related to carbohydrate synthesis were identified in *D. officinale*, of which 430 GTs, 405 GHs, 150 CEs, 77 CBMs and 19 PLs [19]. Compared with *D. officinale*, the number of genes related to carbohydrate synthesis of *D. huoshanens*e was more than that of *D. officinale*, especially in GTs, GHs and PLs. Most of all, the number of GHs in *D. huoshanense* is twice as great as in *D. officinale*. *D. huoshanense* glycoside hydrolysis capacity may be stronger than *D. officinale*. These results indicate that differences in the number and expression levels of carbohydrate-related genes may influence the content of carbohydrates in different *Dendrobium* plants.

Based on previous studies, sucrose plays a central role in the growth and differentiation of plants [46]. Sucrose hydrolysis can be derived from a lot of monosaccharides, and these monosaccharides participate in the synthesis of polysaccharides *D. officinale* [47]. In higher plants, sucrose metabolism is mainly catalyzed by two enzymes: sucrose phosphate synthase (SPS) and sucrose synthase (Susy). In general, the synthesis of sucrose is thought to be catalyzed by SPS, while sucrose decomposition is mainly catalyzed by Susy [48]. In the current market, the quality of *Dendrobium* depends on the content of soluble polysaccharides. One study has shown that soluble polysaccharides and sucrose metabolic enzymes are closely linked, sucrose metabolism is an important part of polysaccharide synthesis [49]. Liang Yan analyzed the genome sequence of *D. officinale*, the essential process of polysaccharide synthesis was obtained, and the key enzymes in sucrose synthesis were identified: 10 *SPS* genes and 15 *Susy* genes. Compared with other species, these genes were in significantly higher expression, suggesting that the expansions of these genes might related to the richness of polysaccharides [21]. We identified 13 *SPS* genes and 18 *Susy* genes in *D. huoshanense*, for the next step to provide the basis for verifying whether these genes are involved in polysaccharides synthesis in *D. huoshanense*.

Plant alkaloids are other active ingredients of *Dendrobium*. In the published *D. officinale* transcriptome data, the alkaloid synthesis pathway of *D. officinale* begins with shikimate pathway and is related to the mevalonate (MVA) pathway or the 2-C-methyl-D-erythritol 4-phosphate (MEP) pathway, but the complete synthesis pathway is not clear [17]. In *D. huoshanense* transcriptome, searching through the KEGG database, 6 key enzyme genes involved in the shikimate pathway and 12 key enzyme genes involved in the MVA or MEP pathway were identified. In the terpenoid pathway, the terpene compound precursors mainly have the mevalonate (MVA) pathway and the 2-C-methyl-D-erythritol 4-phosphate (MEP) pathway; the end products of both pathways are isopentenyl pyrophosphate (IPP). We selected five key enzyme genes (*DXS*, *DXR*, *HDR*, *HMGR* and *FPS*) involved in the MVA and MEP pathways for quantitative validation.

The production processes of the shikimate pathway in plants are not only essential components of protein synthesis, but are also precursors for a wide range of secondary metabolites [50, 51]. EPSP is a shikimate pathway key enzyme involved in the formation of enolpyruvylshikimate 3-phosphate [52]. In *D. huoshanense*, the stem-specific expressed EPSP encoding genes may increase the metabolic rate of shikimate pathway to produce more tryptamines, which are precursors for strictosidine biosynthesis. But the difference is that in *D. officinale*, EPSP encoding genes are the leaf-specific expressed genes, suggest EPSP in leaf-specific accumulation of tryptamine in *D. officinale*. The biosynthesis of secologanin is catalyzed by a series of enzymes associated with MVA and MEP pathways [17]. In the MEP pathway, DXS is the first key enzyme [53], and DXR is the secondary acting and rate limiting enzyme, which catalyzes the formation of straight chain pentose sugars with a branched isovaleric precursor [54]. In *D. huoshanense*, the genes encoding DXS and DXR were mainly expressed in stems. Studies have shown that the excessive expression of DXS in Spike lavender results in a significant increase in terpenoid content [55]; the expression of DXR is enhanced in the hairy roots of *Salvia miltiorrhiza*, which could effectively control and improve the synthesis of terpenoids [56]. We suggest that there is a role of DXS and DXR in stem-specific accumulation of alkaloid in *D. huoshanense*. But in *D. officinale*, they have differences in tissue-specific accumulation of alkaloids, and they are mainly expressed in leaves not in stems [20]. In the MVA pathway, HMGR is the key enzyme in the biosynthesis of plant terpenoids, which is capable of catalyzing 3-hydroxy-3-methylglutaryl coenzyme A (HMG-CoA) to form mevalonate [57]. The overexpression of HMGR1 in ginseng increases the steroids and triterpenes in plants, indicating the effect of promoting ginsenoside biosynthesis [58]. However, the levels of HMGR expressed in stem were partly higher than those in root. This is probably because the biosynthesis of *D. huoshanense* was completed after a multistep biochemical reaction, the catalytic role of HMGR in the upstream, followed by a variety of enzyme synergies that would affect the synthesis of alkaloids. In *D. officinale*, the levels of HMGR expressed in root were partly higher than those in stem. All in all, regarding the synthetic encoding genes expressed in *D. huoshanense* alkaloid, most of genes were expressed highly in stem, but in *D. officinale*, the result was exactly the opposite. They suggested that the *D. officinale* alkaloid was in leaf-specific accumulation.

Following the generation of strictosidine, the alkaloid biosynthesis pathway remains unclear. It is speculated that the downstream synthesis pathway is mainly the modification of strictosidine, which may participate in the oxidation reaction. At present, CYP450 involved in alkaloid biosynthesis remain uncharacterized. Cytochrome P450 (CYP450s), a superfamily of monooxygenase, plays critical roles in biosynthesis of plant secondary metabolites such as triterpenes, alkaloids, and sterols [59]. However, no cytochrome P450 involved in downstream in the alkaloid biosynthetic pathway has been cloned or identified in *Dendrobium* plants. In our study, 229 unigenes were identified as putative P450 superfamily members in *D. huoshanense*. This result is consistent with *D. officinale* [20]. In detail, the largest subfamily (subfamily 71) consists of 18 P450 genes, the second largest subfamily (subfamily 3A) consist of 14 P450 genes and the third subfamily (subfamily 4) consists of 11 P450 genes. CYP71 subfamily was reported to be involved in bioactive secondary metabolism in plants [40]. The CYP3A subfamily of enzymes is the most important to drug metabolism in humans because these enzymes metabolize the majority of commercially available drugs [60]. It is seen from the side that *D. huoshanense* plays a role in drug metabolism in humans, but these are not identified in *D. officinale*. At present, the research on CYP4 subfamily is mainly focused on animals, which mainly metabolize endogenous substances, and some members may also play a role in the metabolism of exogenous substances [61–63]. CYP450 is a major metabolic enzyme for drugs and other endogenous and exogenous substances, which need to be further identified in *Dendrobium* plants.

Through the functional analysis of *D. huoshanense*, the candidate genes related to the synthesis of a large number of polysaccharides and alkaloids were identified, and the sequence information was provided for the gene cloning, gene structure analysis and functional verification of these genes. The results of the study will fill the gaps in functional genomics of *D. huoshanense*, and provide the basis for functional identification, breeding of *D. huoshanense* and optimization of germplasm resources.

## Additional files

Additional file 1: **Table S1.** DEGs associated with alkaloid biosynthesis in *D. huoshanense*. **Table S2.** Putative cytochrome P450s involved in *D. huoshanense* transcriptome. **Table S3.** The information of 47 putative unigenes associated with four independent transaminases. **Table S4.** Genes IDs and primers used in the quantitative real-time PCR (qRT-PCR) experiments. (DOCX 34 kb)

Additional file 2: **Figure S1.** Functional gene ontology classification of unigenes. **Figure S2.** The heatmap of important DEGs associated with glycosyltransferase. **Figure S3** Venn diagram of all unigenes with annotations against five public databases. (DOCX 1469 kb)

Additional file 3: Differential expression genes between Dh_L and Dh_S. (XLS 37782 kb)

Additional file 4: Differential expression genes between Dh_R and Dh_L. (XLS 37941 kb)

Additional file 5: Differential expression genes between Dh_R and Dh_S. (XLS 38011 kb)

Additional file 6: Gene Ontology (GO) enrichment of *D. huoshanense* transcriptome between Dh_L and Dh_S. (XLS 4088 kb)

Additional file 7: Gene Ontology (GO) enrichment of *D. huoshanense* transcriptome between Dh_R and Dh_L. (XLS 4064 kb)

Additional file 8: Gene Ontology (GO) enrichment of *D. huoshanense* transcriptome between Dh_R and Dh_S. (XLS 2487 kb)

Additional file 9: Detailed information about unigenes associated with putative alkaloid biosynthesis in *D. huoshanense*. (XLSX 60 kb)

## Abbreviations

CBMs: Carbohydrate-binding modules
CEs: Carbohydrate esterases
CP: Cellular processes
DD: Drug development
DEGs: Differential expression genes
DHD: 3-dehydroquinate acid dehydratase
DHQS: 3-dehydroquinate synthase
DHS: 3-deoxy-D-arabinoheptulosonate-DHS7-phosphate
EIP: Environmental Information Processing
EPSP: 5-enolpyruvylshikimate-3-phosphate synthase
FC: Fold change
FDR: False discovery rate
FPKM: Fragments per kilobase million
FPS: Famesyl diphosphase synthase
FucTs: Fucosyltransferases
GATs: Galactosyltransferases
GHs: Glycoside hydrolases
GIP: Genetic Information Processing
GO: Gene ontology
GTFs: Glucosyltransferases
GTs: Glycosyltransferase genes
HD: Human diseases
KEGG: Kyoto encyclopedia of genes and genomes
M: Metabolism
MNTs: Mannosyltransferases
Nr: NCBI non-redundant protein sequences
OS: Organismal Systems
Pfam: Protein family
PLs: Polysaccharide lyases
SKDH: Shikimate dehydrogenase
String: Search tool for the retrieval of interacting genes
TDC: Tryptophan decarboxylase
TSB: β-subunit of tryptophan synthase
XTs: Xylosyltransferases
