# Supplementary material for: Analysis of Dendrobium huoshanense transcriptome unveils putative genes associated with active ingredients synthesis
Source: BMC Genomics. 2018 Dec 29;19:978. doi: 10.1186/s12864-018-5305-6 (PMC6310986; doi:10.1186/s12864-018-5305-6)
Supplement: Supplementary file 1 — Table S1. DEGs associated with alkaloid biosynthesis in D. huoshanense. Table S2. Putative cytochrome P450s involved in D. huoshanense transcriptome. Table S3. The information of 47 putative unigenes associated with four independent transaminases. Table S4. Genes IDs and primers used in the quantitative real-time PCR (qRT-PCR) experiments. (DOCX 34 kb) [file 12864_2018_5305_MOESM1_ESM.docx]

**Additional file 1**

**Table S1** **DEGs associated with alkaloid biosynthesis in *Dendrobium huoshanense***

| **EC** | **Definition** | **Number of DEGs (Dh_L vs. Dh_S)** | | **Number of DEGs (****Dh_R vs. Dh_L)** | | **Number of DEGs (Dh_R vs. Dh_S)** |
| --- | --- | --- | --- | --- | --- | --- |
| 2.5.1.54 | 3-deoxy-D-arabinoheptulosonate-DHS7-phosphate (DHS) | 16 | | 16 | | 7 |
| 4.2.3.4 | 3-dehydroquinate synthase (DHQS) | 11 | | 3 | | 6 |
| 4.2.1.10 | 3-dehydroquinate acid dehydratase (DHD) | 8 | | 3 | | 3 |
| 1.1.1.25 | shikimate dehydrogenase (SKDH) | 6 | | 2 | | 2 |
| 2.7.1.71 | shikimate kinase（SK） | 7 | | 3 | | 2 |
| 2.5.1.19 | 5-enolpyruvylshikimate-3-phosphate synthase (EPSP) | 5 | | 2 | | 1 |
| 2.3.1.9 | Acetyl-CoA acetyltransferase (AACT) | 4 | | 4 | | 4 |
| 2.3.3.10 | 3-hydroxy-3-methylglutaryl coenzyme A synthase (HMGS) | 8 | | 2 | | 2 |
| 1.1.1.34 | 3-hydroxy-3-methylglutaryl coenzyme A reductase (HMGR) | 4 | | 5 | | 5 |
| 2.7.1.36 | Mevalonate kinase (MVK) | 2 | | 1 | | 1 |
| 2.7.4.2 | Phosphomevelonate kinase (PMK) | | 1 | | 0 | 0 |
| 4.1.1.33 | Mevalonate diphosphase decarboxylase (MVD) | | 2 | | 0 | 1 |
| 2.5.1.10 | Famesyl diphosphase synthase (FPS) | | 10 | | 6 | 7 |
| 5.3.3.2 | Isopentenyl diphosphate isomerase (IPI) | | 6 | | 0 | 4 |
| 2.2.1.7 | 1-deoxyxylulose-5-phosphate synthetase (DXS) | | 6 | | 4 | 6 |
| 1.1.1.267 | 1-deoxy-D-xylulose-5-phosphate reductoisomerase (DXR) | | 2 | | 2 | 2 |
| 2.7.7.60 | 4-diphosphocytidyl-2C-methyl-D-erythritol synthase (CMS) | | 2 | | 0 | 1 |
| 2.7.1.148 | 4-diphosphocytidyl-2C-methyl-D-erythritol kinase (CMK) | | 3 | | 0 | 2 |
| 4.6.1.12 | 2-C-methyl-D-erythritol 2,4-cyclodiphosphate synthase (MCS) | | 3 | | 1 | 1 |
| 1.17.7.1 | 4-hydroxy-3-methylbut-2-enyl diphosphate synthase (HDS) | | 5 | | 1 | 2 |
| 1.17.7.4 | 4-hydroxy-3-methylbut-2-enyl diphosphate reductase (HDR) | | 2 | | 1 | 1 |
| Total number | | | 113 | | 56 | 60 |

**Table S2 Putative cytochrome P450s involved in *D. huoshanense* transcriptome.**

| Cytochrome P450s family member | No. of unigenes |
| --- | --- |
| Cytochrome P450 1A1 | 1 |
| Cytochrome P450 2E1 | 1 |
| Cytochrome P450 2J2 | 1 |
| Cytochrome P450 2K1 | 1 |
| Cytochrome P450 20A1 | 1 |
| Cytochrome P450 24A1 | 2 |
| Cytochrome P450 3A11 | 1 |
| Cytochrome P450 3A12 | 2 |
| Cytochrome P450 3A2 | 3 |
| Cytochrome P450 3A21 | 1 |
| Cytochrome P450 3A24 | 1 |
| Cytochrome P450 3A31 | 1 |
| Cytochrome P450 3A7 | 2 |
| Cytochrome P450 3A9 | 1 |
| Cytochrome P450 3A4 | 1 |
| Cytochrome P450 30A1 | 1 |
| Cytochrome P450 3045C4 | 2 |
| Cytochrome P450 313A2 | 1 |
| Cytochrome P450 4A10 | 3 |
| Cytochrome P450 4A11 | 1 |
| Cytochrome P450 4B1 | 2 |
| Cytochrome P450 4C1 | 1 |
| Cytochrome P450 4C3 | 1 |
| Cytochrome P450 4E3 | 1 |
| Cytochrome P450 4F6 | 1 |
| Cytochrome P450 4Z1 | 1 |
| Cytochrome P450 5008A2 | 1 |
| Cytochrome P450 5010A1 | 1 |
| Cytochrome P450 51 | 2 |
| Cytochrome P450 52A1 | 1 |
| Cytochrome P450 52A11 | 3 |
| Cytochrome P450 52A12 | 4 |
| Cytochrome P450 52A13 | 1 |
| Cytochrome P450 55A2 | 2 |
| Cytochrome P450 55A3 | 1 |
| Cytochrome P450 61 | 2 |
| Cytochrome P450 704C1 | 4 |
| Cytochrome P450 706A6 | 1 |
| Cytochrome P450 711 | 3 |
| Cytochrome P450 71A1 | 6 |
| Cytochrome P450 71A2 | 1 |
| Cytochrome P450 71A9 | 1 |
| Cytochrome P450 71D11 | 1 |
| Cytochrome P450 71D13 | 3 |
| Cytochrome P450 71D7 | 1 |
| Cytochrome P450 714B3 | 2 |
| Cytochrome P450 71D95 | 3 |
| Cytochrome P450 72A15 | 1 |
| Cytochrome P450 72A219 | 4 |
| Cytochrome P450 72A26 | 1 |
| Cytochrome P450 734A1 | 2 |
| Cytochrome P450 749A22 | 1 |
| Cytochrome P450 76C3 | 1 |
| Cytochrome P450 77A2 | 1 |
| Cytochrome P450 77A3 | 1 |
| Cytochrome P450 78A3 | 1 |
| Cytochrome P450 78A4 | 1 |
| Cytochrome P450 78A5 | 5 |
| Cytochrome P450 81 | 2 |
| Cytochrome P450 84A1 | 1 |
| Cytochrome P450 85A1 | 2 |
| Cytochrome P450 86A8 | 1 |
| Cytochrome P450 86B1 | 4 |
| Cytochrome P450 87A3 | 2 |
| Cytochrome P450 89A1 | 2 |
| Cytochrome P450 89A2 | 2 |
| Cytochrome P450 9B2 | 1 |
| Cytochrome P450 90B1 | 4 |
| Cytochrome P450 90D2 | 2 |
| Cytochrome P450 93A2 | 1 |
| Cytochrome P450 94A1 | 1 |
| Cytochrome P450 94C1 | 3 |
| Cytochrome P450 98A2 | 1 |
| Cytochrome P450 99A2 | 1 |
| Cytochrome P450 C-22 desaturase | 2 |
| Cytochrome P450 reductase | 58 |
| Cytochrome P450 monooxygenase | 41 |
| Total | 229 |

**Table S3 The information of 47 putative unigenes associated with four independent transaminases.**

| **Name** | **Unigene ID** |
| --- | --- |
| **Alanine, aspartate and glutamate metabolism** | c376623_g1 |
|  | c80188_g1 |
|  | c421993_g1 |
|  | c378946_g1 |
|  | c384214_g1 |
|  | c377479_g2 |
|  | c253687_g1 |
|  | c385719_g1 |
|  | c362321_g1 |
|  | c408239_g3 |
|  | c408239_g2 |
|  | c408239_g1 |
| **Porphyrin and chlorophyll metabolism** | c351157_g1 |
|  | c412367_g1 |
|  | c420539_g1 |
| **Glycine, serine and threonine metabolism** | c404135_g1 |
|  | c384723_g1 |
|  | c419000_g1 |
|  | c394161_g1 |
|  | c419000_g2 |
|  | c383041_g1 |
|  | c388256_g1 |
|  | c422192_g2 |
|  | c422192_g3 |
|  | c371396_g2 |
|  | c422192_g1 |
|  | c360307_g1 |
|  | c404483_g2 |
|  | c381016_g1 |
|  | c404483_g1 |
|  | c371396_g1 |
|  | c403165_g1 |
|  | c403098_g1 |
| **Valine, leucine and isoleucine degradation** | c420450_g5 |
|  | c415396_g1 |
|  | c391814_g1 |
|  | c404778_g2 |
|  | c420450_g4 |
|  | c420450_g3 |
|  | c420450_g2 |
|  | c388903_g1 |
|  | c410286_g3 |
|  | c301759_g2 |
|  | c380472_g1 |
|  | c401262_g1 |
|  | c311700_g1 |
|  | c784947_g1 |

**Table S4** **Genes IDs and primers used in the quantitative real-time PCR (qRT-PCR) experiments.**

| Primer_ID | Forward PCR Primer (5′-3′) | Reverse PCR Primer (5′-3′) |
| --- | --- | --- |
| *Actin* | CGTGGAGGAGACTCGTGAGG | CGATGCCTGGCGACACTTTC |
| c406740_g1 (*HMGR*) | ACATCAGCCTGCTCGAGACC | ACTTCCAGGCCGTCTGATCG |
| c418084_g7 (*HMGR*) | CGACGCCCTTCCAATCCCTA | GCGGCGCATGAGAAAGACAA |
| c301772_g1 (*HMGR*) | AGCGTTCTGAAGACGACGGT | GCCAAGAACACCGCAGTGAG |
| c368929_g1 (*HMGR*) | GGTGCTATTGGTGCTAGTTGCC | CATTCAGCAGCACAAGCTGGA |
| c282476_g1 (*HMGR*) | AGGCTATCGCCATGCTCACA | GGTCGTTGAGCTCGCTCTCT |
| c343028_g1 (*FPS*) | CCTCAAGCAGGCAGAGGACA | GGTTGACCAACCACGAGCAC |
| c402550_g1 (*FPS*) | CAGGAGGGAAGCTCAACCGT | GCCTGAAGCCATTCAACACACC |
| c387734_g1 (*FPS*) | GAGGCCAGGGCATGGATCTT | CGTAGGACTAGGGCCGTTGG |
| c210849_g1 (*FPS*) | AGGCTGCCAGTCTTCTGCTC | GTTGCCTACCGCTCCGTACT |
| c387496_g1 (*FPS*) | TGGAGGTCGAGCGTTTGAGG | CCCAGCTCCTCCGACGATTT |
| c410441_g3 (*FPS*) | TCGGATGTACTGGGCCAACC | ACCTCATCAGCCAGGCCATC |
| c379694_g1 (*FPS*) | CGCTCTCGAGATGGTCCACA | AGCGAGATCGACGCCAAAGA |
| c382607_g2 (*DXS*) | TGCTGCATGGTGTACTGGCT | AGGCGCGACAGAACTGAGAA |
| c385678_g1 (*DXS*) | CGGGACAGGATGCACACCAT | CTTGAAGTCACGCCCAACCG |
| c383947_g1 (*DXS*) | TGGCCTCCCTCCATCGAGTA | GGGCAGAAGAGGGCAAGGAA |
| c382607_g1 (*DXS*) | TCAAGGCGACGGAGACAGTG | GCTTTCCTGTCAGCGTGTCG |
| c415809_g2 (*DXR*) | CCATCCGAGAGGGCAAGGAC | GATGGCAGAGTGCTCCGAGT |
| c415809_g1 (*DXR*) | CGACTCAGAGCACTCTGCCA | CAGCTTCTCCACAGGCCACT |
| c421755_g2 (*HDR*) | CAGGTCATGCCCGCCTAGAA | AGCTGCCAGAAGCTGAGAGG |
| c353140_g1 (*HDR*) | TTGGTCAGGCGCTTGGTGTA | AGGGTGAGGCCAAGAAGCTG |
