# Supplementary material for: Analysis of Dendrobium huoshanense transcriptome unveils putative genes associated with active ingredients synthesis
Source: BMC Genomics. 2018 Dec 29;19:978. doi: 10.1186/s12864-018-5305-6 (PMC6310986; doi:10.1186/s12864-018-5305-6)
Supplement: Supplementary file 2 — Figure S1. Functional gene ontology classification of unigenes. Figure S2. The heatmap of important DEGs associated with glycosyltransferase. Figure S3 Venn diagram of all unigenes with annotations against five public databases. (DOCX 1469 kb) [file 12864_2018_5305_MOESM2_ESM.docx]

Additional file 2


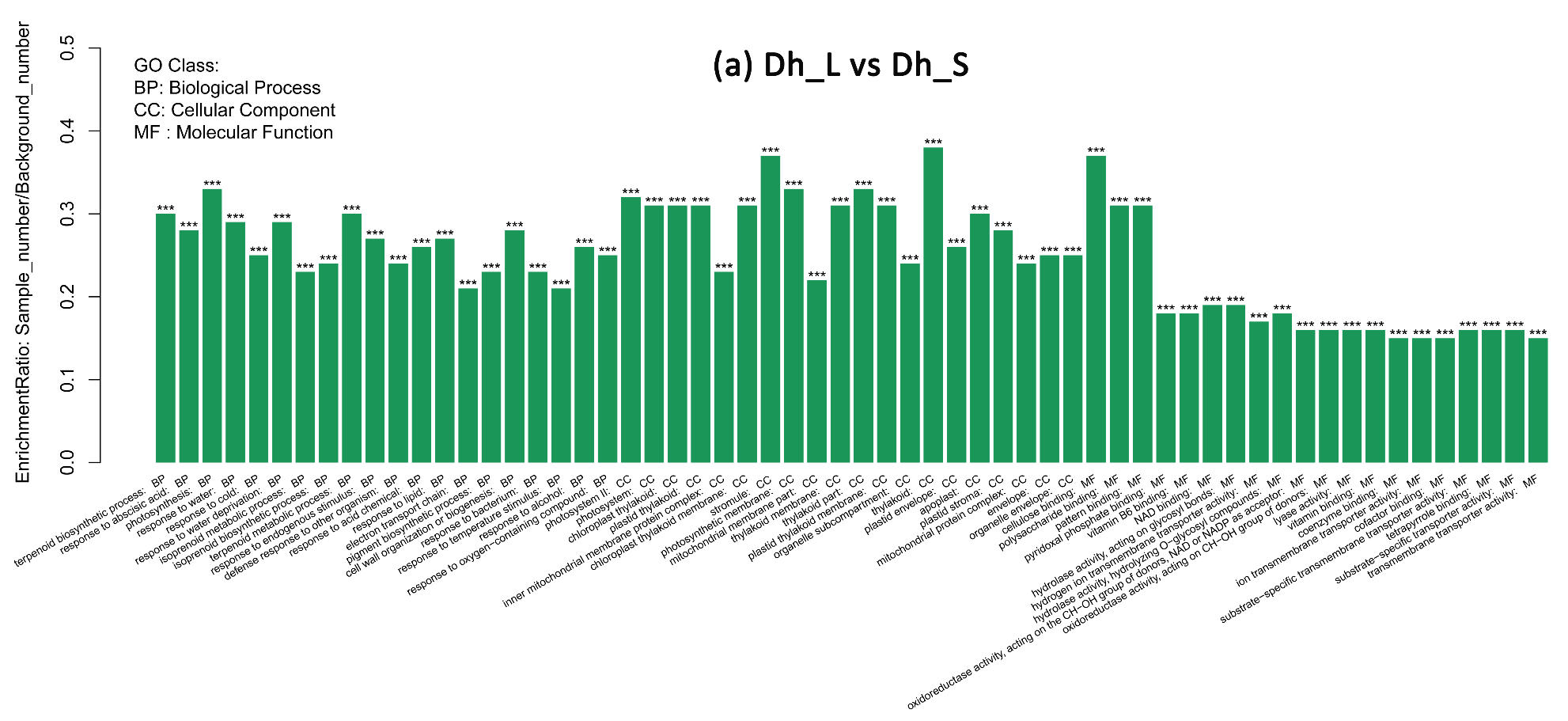


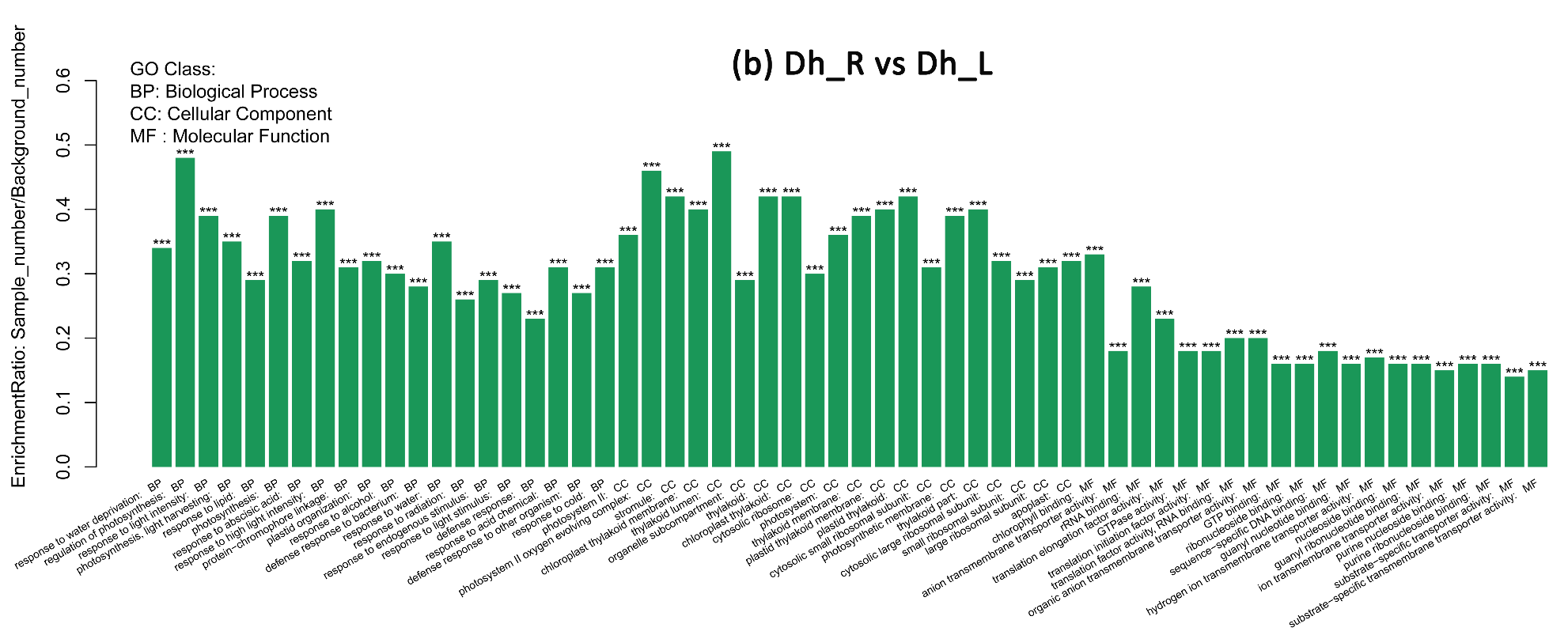

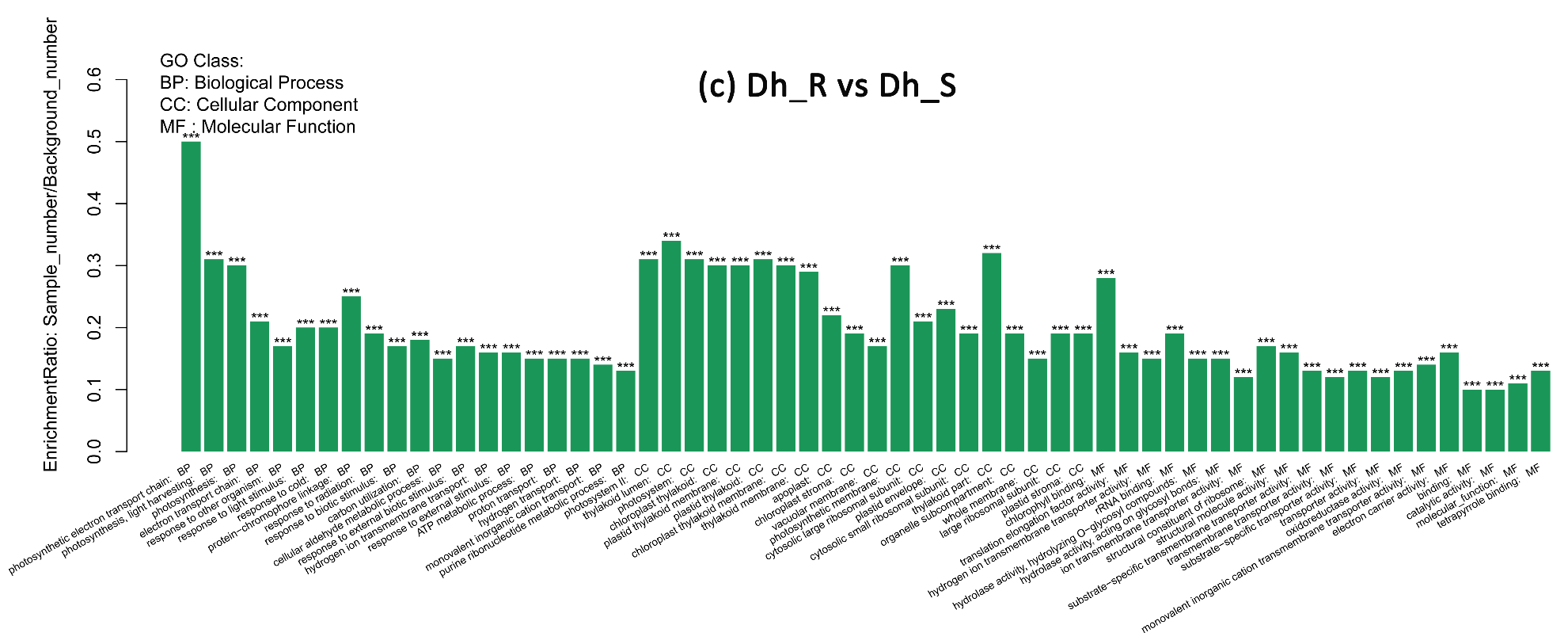


**Supplementary FIGURE S1** **Functional gene ontology (GO) enrichment of DEGs.** The y-axis indicates the enrichment ratio of unigenes in a category. (a) Dh_L vs. Dh_S; (b) Dh_R vs. Dh_L; (c) Dh_R vs. Dh_S. All pathways in the figure indicate significant GO enrichment, with three asterisks indicating FDR＜0.001.


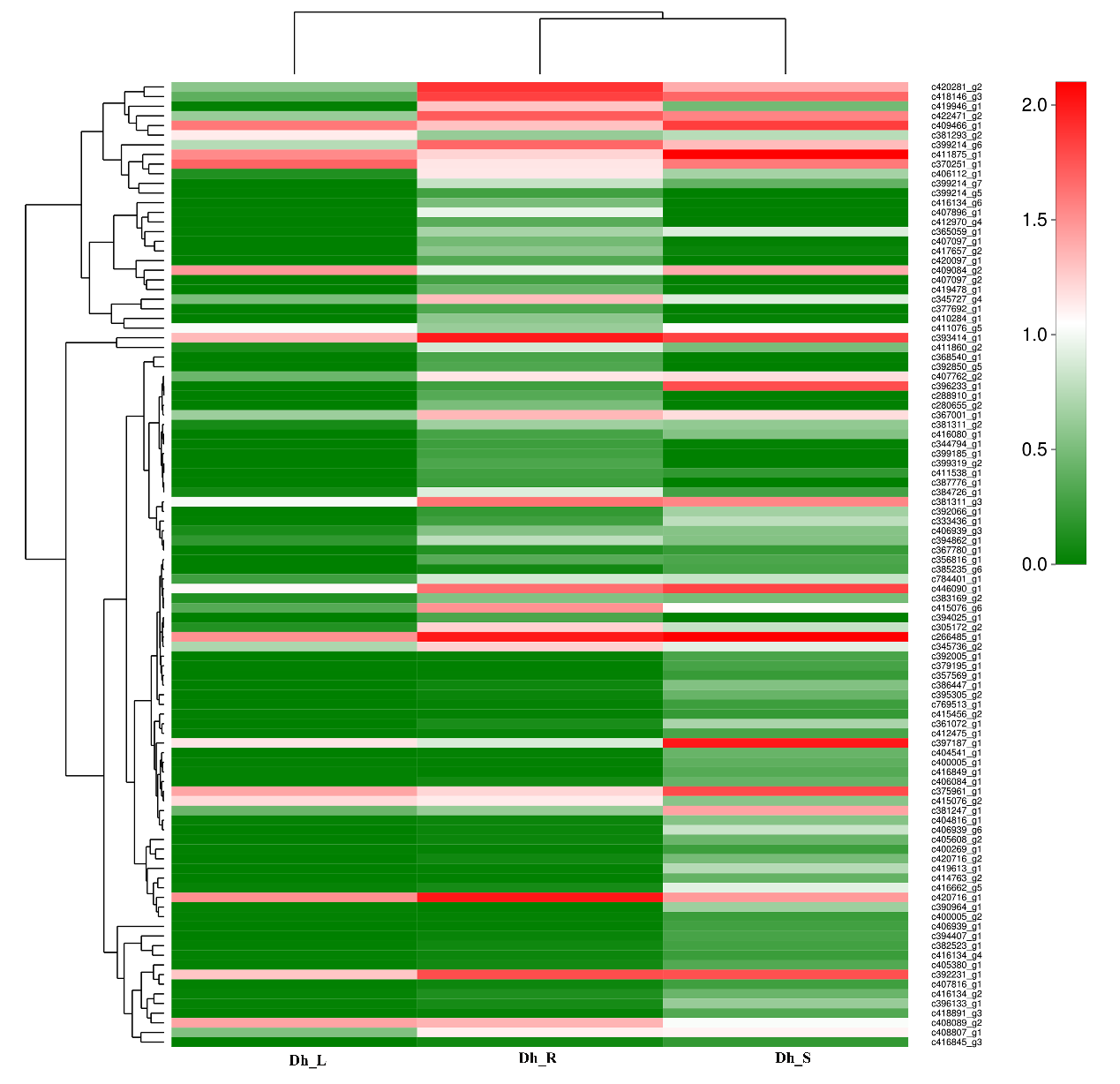


**Supplementary FIGURE S2** **The heatmap of important DEGs associated with glycosyltransferase.** Red indicates high expression genes, while green indicates low expression genes. Color from red to green indicate that log_10_ (FPKM+1) gradually changes from big to small.


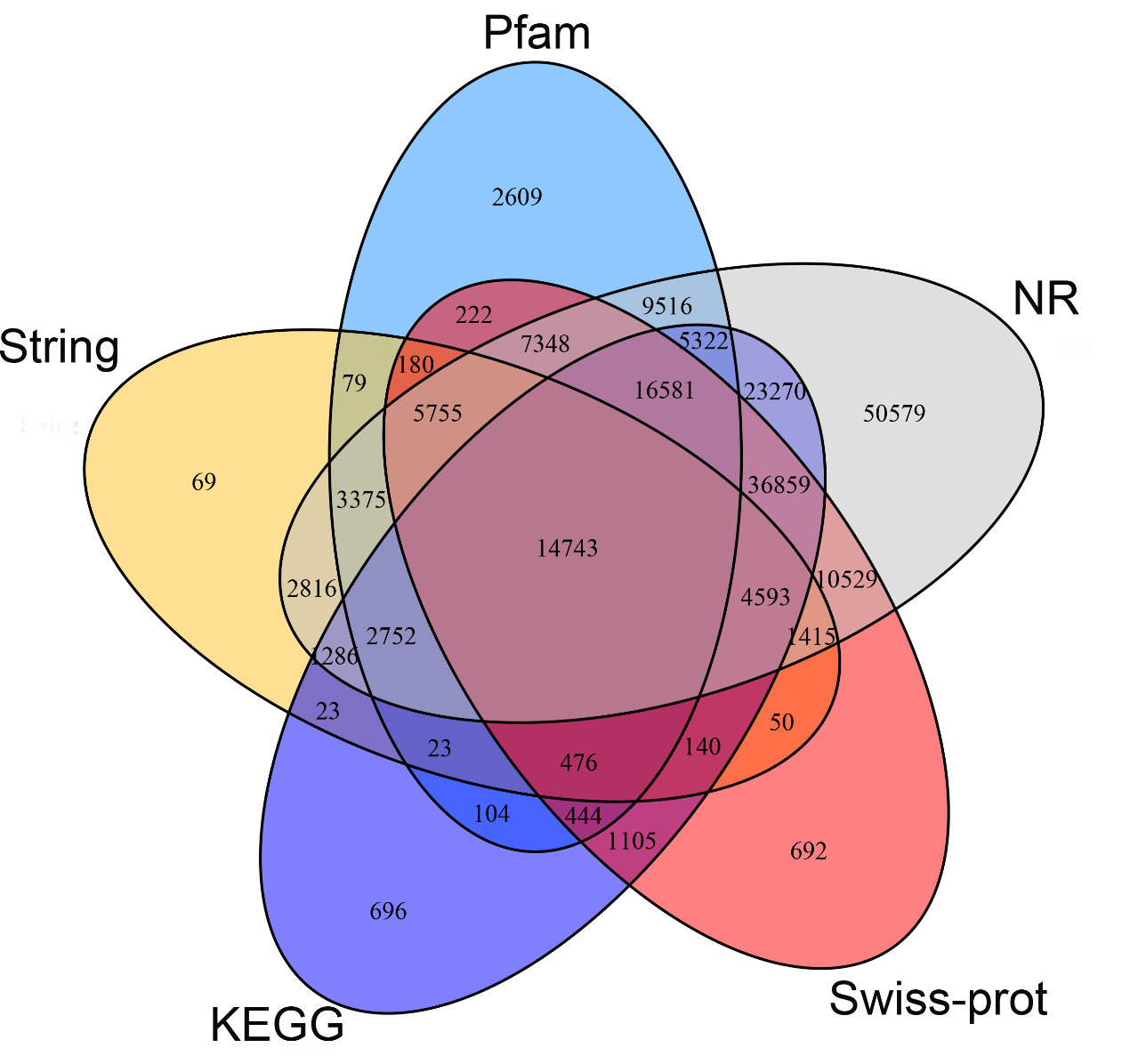


**Additional file 1 FIGURE S3 Venn diagram of all unigenes with annotations against five public databases**
